# Rate Constants and Energetics of the H_2_SiO + H Reaction System: RP‐VTST/MT and VRC Calculations

**DOI:** 10.1002/jcc.70115

**Published:** 2025-05-19

**Authors:** Marcelo André Petry Pontes, Edson Firmino Viana de Carvalho, Luiz Fernando de Araujo Ferrão, Francisco Bolivar Correto Machado, Orlando Roberto‐Neto

**Affiliations:** ^1^ Departamento de Química Instituto Tecnológico da Aeronáutica São José dos Campos São Paulo Brazil; ^2^ Laboratório de Computação Científica Avançada e Modelamento (Lab – CCAM), Instituto Tecnológico da Aeronáutica São José dos Campos São Paulo Brazil; ^3^ Departamento de Engenharia Faculdade Anhanguera de Jacareí Jacareí São Paulo Brazil; ^4^ Departamento de Física Universidade Federal Do Maranhão São Luís Maranhão Brazil; ^5^ Divisão de Aerotermodinâmica e Hipersônica Instituto de Estudos Avançados São José dos Campos São Paulo Brazil

**Keywords:** combustion of silane, computational kinetics, hydrogen abstraction reaction, potential energy surface, silaformaldehyde

## Abstract

Silaformaldehyde (H_2_SiO) is one of the components of the kinetics roadmap of silane oxidation. For this species, kinetics decomposition is related to three elementary reactions, that is, H + H_2_SiO → H_2_ + HSiO (R1), H + H_2_SiO → H_2_SiOH (R2), and H + H_2_SiO → H_3_SiO (R3). To improve the kinetics of these reaction systems, accurate energetics were computed with the ωB97X‐D and CCSD(T) methods, and the rate constants were determined using CVT methods with multidimensional tunneling. KIEs were also determined for (R1), which is an important path at high temperatures. At the ωB97X‐D/aug‐cc‐pVTZ level, the value of electronic barrier height is 4.5, 5.2, and 0.4 kcal mol^−1^ for (R1), (R2), and (R3), respectively. In addition to the characterization of the elementary reactions, a mechanism consisting of all interconnected reactions was characterized by using the energy‐grained master equation approach to determine the phenomenological rate constants for the formation of products and the time evolution of the species. Up to 500 K, the main reaction product is H_2_SiOH, while the bimolecular products H_2_ + HSiO dominate at higher temperatures.

## Graphical abstract

The decomposition of Silaformaldehyde (H_2_SiO) involves three reactions: H + H_2_SiO → H_2_ + HSiO (R1), H + H_2_SiO → H_2_SiOH (R2), and H + H_2_SiO → H_3_SiO (R3). Accurate energetics were calculated using ωB97X‐D and CCSD(T), and rate constants using CVT methods with multidimensional tunneling. Product formation and species evolution were modeled using the master‐equation approach.

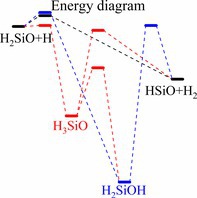

## 1 Introduction

Silane (SiH_4_) was first synthesized by Wohler and Buff [1], and thenceforward, it has been used in the manufacture of semiconductor films, photovoltaic panels, and electronic devices [2, 3]. In addition to its critical role in materials science, silane has been applied as an additive fuel for aerospace propulsion [4, 5, 6]. Consequently, the validation of kinetic models of silane oxidation and pyrolysis is of great importance. This endeavor requires the determination of accurate values of thermochemical and kinetic parameters within a temperature range of medium‐to‐high pressure and temperatures [7, 8, 9].

In earlier investigations, Jachimowski and MacLain [10] and Chinitz [11] proposed a chemical reaction mechanism for the combustion of SiH_4_/O_2_/H_2_ based on the kinetics and thermochemical parameters of the CH_4_/O_2_/H_2_ combustion. This was subsequently refined through shock tube measurements conducted within the temperature range of 800–1200 K. Britten et al. [12] have developed an improved kinetic model of combustion, comprising a system of 70 elementary reaction steps and 25 chemical compounds.

The discrepancies observed in the comparison of silane combustion models [6, 7, 8, 9] indicate the necessity of new, more accurate measurements to improve the predictive capabilities of silane/H_2_ models [6]. For instance, the results of collision limit violations (CLVs) analysis and G4 calculations [7] demonstrate a significant divergence between the experimental determination of the enthalpy of formation of H_2_SiO (silaformaldehyde, the simplest silanone).

One of the elementary reactions of silane combustion that presents a lack of accurate thermochemical and chemical kinetic data is the abstraction reaction of H_2_SiO by H (R1), resulting in the formation of HSiO and H_2_ as products. Also, there are the H_2_SiOH (R2) and H_3_SiO (R3) involved in parallel reactions of elimination of H_2_SiO [13],

(R1) $$H+H_{2}\mathrm{SiO}\to \text{HSiO}+H_{2}$$

(R2) $$H+H_{2}\mathrm{SiO}\to H_{2}\text{SiOH}$$

(R3) $$H+H_{2}\mathrm{SiO}\to H_{3}\mathrm{SiO}$$

Zachariah and Tsang [13] have employed the HF/6‐31G(d) and MP2/6‐31G(d) methods to compute the geometries and vibrational frequencies of reactants, products, and the transition state of (R1). The classical barrier height and enthalpy of reaction at 298 K were computed with the bond additivity correction (BAC) method and using HF‐SCF and MP2 geometries, resulting in an activation energy of 0.31 kcal mol^−1^ [13]. The recommended three‐parameter expression is *k*_1_(*T*) = 2.44 × 10^−11^ (cm^3^ molecule^−1^) (*T*/298 K)^0.58^ × exp.[(−30.26 kJ mol^−1^)/(*RT*)] and *k*_2_(*T*) = 1.34 × 10^−8^ (cm^3^ molecule^−1^) (*T*/298 K)^−3.6^ × exp.[(−34.4 kJ mol^−1^)/(*RT*)] obtained from RRKM extrapolated data to the temperature range of 1000–2500 K [13].

The main goal of this work is to improve the accuracy of the electronic barrier height and reaction energy calculations of ((R1), (R2), (R3)), which are well‐known critical parameters in chemical dynamics, and the computation of the accurate thermal rate constants utilizing the reaction‐path variational transition state theory with multidimensional tunneling (RP‐VTST/MT) [14], or variable‐reaction‐coordinate variational transition state theory (VRC‐VTST) [15, 16]. A secondary interest of this investigation was the chemical kinetics study at moderate to high temperatures and pressures, the reason for possible applications in scramjet ignition [6, 13].

Given the exoergicity of (R2) and the low barrier height of (R3), three subsequent steps were taken into consideration starting from the products of (R2) and (R3):

(R2‐1) $$H_{2}\text{SiOH}\to \text{HSiO}+H_{2}$$

(R3‐1) $$H_{3}\mathrm{SiO}\to \text{HSiO}+H_{2}$$

(R3‐2) $$H_{3}\mathrm{SiO}\to H_{2}\text{SiOH}$$

Also, since there are no previous experimental or theoretical studies of reactions involving the deuterium isotopomers, with the exception of crossed molecular beams data of D_2_SiO [17], we have carried out calculations of kinetic isotope effects (KIEs) (*k* ^H^/*k* ^D^) for the dominant reaction path (R1) considering one of the reactants as deuterated.

Complementary to the characterization of isolated elementary reactions, a mechanism consisting of all interconnected reactions (R1), (R2), (R3), (R2‐1), (R3‐1), and (R3‐2) was studied using an energy‐grained master equation (EGME) approach to take into account the effects of multiple wells, submerged barrier heights, and recrossing reactions to determine the phenomenological rate constants for the formation of products and time evolution of the species.

## 2 Methodology

The equilibrium geometries and harmonic vibrational frequencies of the stationary points (i.e., reactants, products, and saddle point) of the reaction paths (R1), (R2), (R3), (R2‐1), (R3‐1), and (R3‐2) were computed with the M06‐2X [18], and ωB97X‐D [19], density functional theory (DFT) approximations, and the coupled‐cluster with single‐double and perturbative triples (CCSD(T)) method [20], with the correlation consistent aug‐cc‐pVTZ basis sets [21]. The energetic properties calculated are the classical barrier height ($V^{‡}$, electronic energy difference between the reactants and the saddle point), the adiabatic barrier height ($∆V_{a,v}^{‡}$ defined as $V^{‡}$ + ΔZPE), the electronic energy of the reaction (Δ*E*), and the enthalpy of the reaction at 0 K (${\mathrm{\Delta H}}_{x}^{0}=∆E+∆\mathrm{ZPE}$) [22].

To improve the values of the energies, single‐point CCSD(T) calculations were carried out with the aug‐cc‐pV5Z basis set [21] using the CCSD(T)/aug‐cc‐pVTZ geometries (CCSD(T)/aug‐cc‐pV5Z//CCSD(T)/aug‐cc‐pVTZ).

To verify the multireference character of the wave function of reactants, transition state (saddle point), and products, the *T*_1_ diagnostic [23] was employed. The *T*_1_ diagnostic is defined as the Euclidean norm of the *T*_1_ amplitudes vector in a CCSD wave function. It has been proposed that values larger than 0.020 for closed‐shell and larger than 0.045 for open‐shell [24] systems are indicative of the multireference character of the electronic wave function. The electronic structure calculations were carried out with GAUSSIAN 09 quantum chemistry code [25].

The thermal rate constants of hydrogen abstraction reactions (R1) and (R2) were calculated with the RP‐VTST method within the canonical ensemble approach [14]. For that, the electronic minimum energy path (MEP) and the vibrational‐adiabatic potential energy ($∆V_{a,V}^{‡}$) were computed using the dual‐level direct dynamics methodology, using the ωB97X‐D [19], for the electronic structure calculations of the low‐level potential energy surface (PES). The CCSD(T)/aug‐cc‐pV5Z and CCSD(T)/aug‐cc‐pVTZ approaches are respectively employed as the high‐level for the electronic energies and the vibrational frequencies at reactants, products, and saddle points. This chemical kinetics model is known as variational transition state theory with interpolated optimized corrections (VTST‐IOCs) [26]. The MEPs were calculated in isoinertial coordinates from 0.5 *a*_0_ (reactants side) to −3.0 *a*_0_ (products side) for reactions (R1) and (R2). The step size employed was 0.02, and the Hessians were computed every five steps. The reduced mass *μ* was set as 1 amu. The Page–McIver steepest‐descent stabilization [27] and the RODS (reorient the dividing surface) [28] methods were also employed to improve the convergence of the reaction path.

The canonical VTST rate constants for bimolecular reaction (*k* ^CVT^) rate at temperature *T*, which locates the dividing surface along the reaction coordinate (*s*), are given by [29],

(1) $$k^{\mathrm{CVT}}\left(T\right)=\sigma \frac{k_{B}T}{h}\frac{Q^{\mathrm{GT}}\left(T,s_{*}^{\mathrm{CVT}}\left(T\right)\right)}{\Phi_{R}\left(T\right)}\exp\left[-V_{\mathrm{MEP}}\left(T,s_{*}^{\mathrm{CVT}}\left(T\right)\right)/k_{B}T\right]$$

where *σ* is the reaction‐path symmetry number, *k*_B_ and *h* are the Boltzmann and Planck constants, respectively. The values of σ for the forward path were calculated according to the recommendation of Fernández‐Ramos et al. [30]. For H, H_2_SiO, and TS, the assumed Point Group and rotational symmetry numbers (σ_rot_) (in parentheses) are, respectively, (C_∞v_, 1), (C_2v_, 2), and (C_s_, 1), and the forward reaction value of symmetry σ is 2. *V*_MEP_ is the value of the potential on the reaction path at $s_{*}^{\mathrm{CVT}}\left(T\right)$, which is the location along the reaction coordinate of the dividing surface that minimizes the one flux rate constant. The quantized reactant partition function per unit volume is Φ_R_ (T), and Q^GT^ (T, $s_{*}^{\mathrm{CVT}}\left(T\right)$) is the quantized generalized transition state partition function at $s_{*}^{\mathrm{CVT}}\left(T\right)$.

Quantum effects on the reaction coordinate are incorporated by multiplying the CVT rate constants with a transmission coefficient, *κ* ^CVT/SAG^, and the resulting rate constants are given by

(2) $$k^{\mathrm{CVT}/\mathrm{SAG}}\left(T\right)=\kappa^{\mathrm{CVT}/\mathrm{SAG}}\left(T\right)k^{\mathrm{CVT}}\left(T\right)$$

where SAG represents the semiclassical vibrationally adiabatic ground state. The transmission factor *κ* ^SAG^(*T*) can be written as

(3) $$\kappa^{\mathrm{SAG}}\left(T\right)=\beta \;\exp\left(\beta V_{a}^{\mathrm{AG}}\right)\int_{0}^{\infty }dE\;P^{\mathrm{SAG}}\left(E\right)\;\exp\left(-\mathrm{\beta E}\right)$$

where $\beta =\frac{1}{k_{B}T}$, $V_{a}^{\mathrm{AG}}$ represents the vibrationally adiabatic ground‐state potential energy, and $P^{\mathrm{SAG}}\left(E\right)$ is the energy‐dependent probability function within the SAG approximation.

The transmission coefficients employed are calculated using a variety of methods, including multidimensional zero‐curvature tunneling (ZCT) [31] and small‐curvature tunneling (SCT) [32, 33].

Reaction paths with low or no barrier (R3) were computed using the canonical (CVT), microcanonical (μVT), and energy and total angular momentum resolved (*E,J*‐μVT) ensembles within the VRC formalism [15, 16]. In VRC, the reaction coordinate and dividing surfaces are defined in a different way than in RP‐VTST. The reaction coordinate *s* is defined by the distance between a pivot point on one reactant and that on another, and the dividing surfaces are defined in terms of pivot points tied to each of the reactants. Different numbers and locations of pivot points lead to different reaction coordinates and dividing surfaces, and the reactive flux is variationally minimized with respect to both the number and position of pivots and the distance between the pivot points in the two reactants. For (R3), the pivot points of the H_2_SiO molecule are positioned ±0.5 a.u. from the Si atom, with the two vectors connecting the pivot points and the Si atom perpendicularly oriented to the H_2_SiO *xy*‐plane. Another point is in the center of the H radical. In the VRC‐VTST calculation, the reaction coordinate(s) is defined as the shortest distance between one of the two pivot points in the H_2_SiO molecule (+0.5 or −0.5 a.u. of the *z*‐axis) and the pivot point 3 in the H radical. This leads to the creation of a two‐faceted dividing surface. A series of *s* values ranging from 3.4 to 6.0 a.u., in increments of 0.1 a.u., were examined to minimize the rate constants with respect to the *s* coordinate. For each dividing surface, 500 configurations were sampled using Monte Carlo integration [34], resulting in a total of 1000 single‐point energy calculations.

All RP and VRC rate constants are computed with the POLYRATE 2023 software package [35]. The computer codes for performing reaction rate constant calculations with VTST‐IOC and VRC have been incorporated into a software package called GAUSSRATE [36], which provides a versatile program interface between GAUSSIAN and POLYRATE.

Complementary to the RP and VRC calculations, RRKM rate constants corrected with Eckart tunneling [37] were also determined using the energetics of the stationary states obtained at the ωB97X‐D/aug‐cc‐pVTZ level. These data were used in an EGME approach to determine the phenomenological rate constants for the formation of products and time evolution of the species, considering a mechanism containing all the studied elementary reactions (R1), (R2), (R3), (R2‐1), (R3‐1), and (R3‐2). The EGME approach was carried out using the MESMER software [38], using quad‐double arithmetic precision. Within the exponential down collisional energy transfer model, the average energy transferred per collision parameter was set to 230 cm^−1^, while the grain size was set to 100 cm^−1^. Argon was used as a thermal bath gas, and the concentration of the excess reactant (H_2_SiO) was set to 1.0% of the bath gas.

## 3 Results and Discussion

### 3.1 Molecular Structures and Energetics

The multireference character of the reactants, transition states, and products for (R1) was verified by the *T*_1_ diagnostic [23]. The CCSD(T)/aug‐cc‐pVTZ geometries were utilized to determine the *T*_1_ diagnostic (and the results are collected in the Supporting Information S1), which indicates that all species, including saddle points and products, are within the limit of 0.044 recommended by Rienstra‐Kiracofe et al. [24].

**FIGURE 1:**
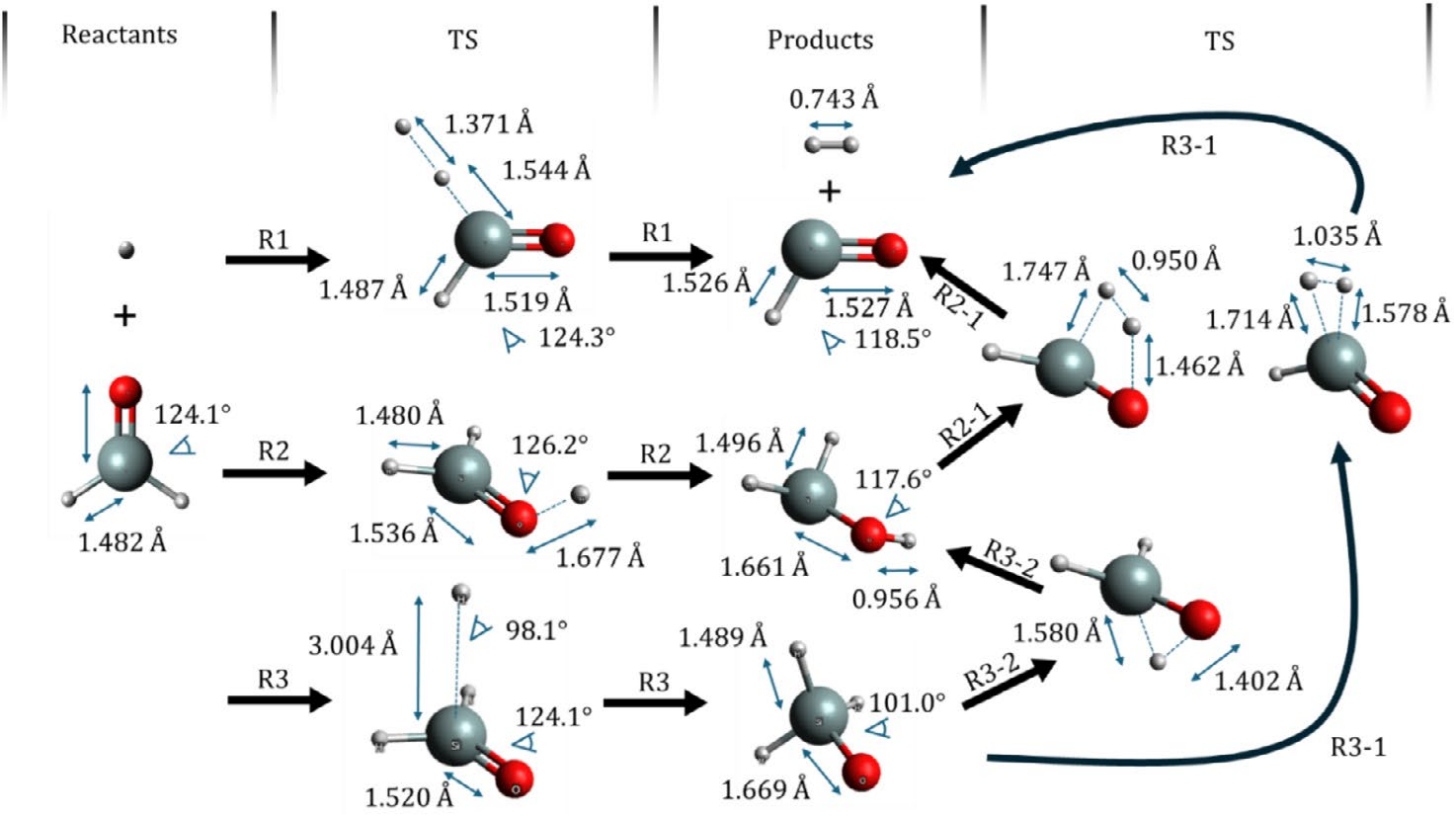
The equilibrium structures of the stationary states of the H + H_2_SiO reaction system computed with the ωB97X‐D/aug‐cc‐pVTZ.

The equilibrium geometries of all species, computed with ωB97X‐D using the aug‐cc‐pVTZ basis set, are represented in Figure 1, including some selected geometric parameters. There is a general agreement between the structures obtained with CCSD(T) and ωB97X‐D. The exception goes to the saddle point of (R3), which is found only at the ωB97X‐D level and presents a long C–H bond length with a shallow barrier. The Cartesian coordinates of the optimized geometries, electronic energies, and zero‐point corrected electronic energies (ZPVE) of reactants, products, and transition states computed with all methods and basis sets are provided in the Supporting Information S1.

**FIGURE 2:**
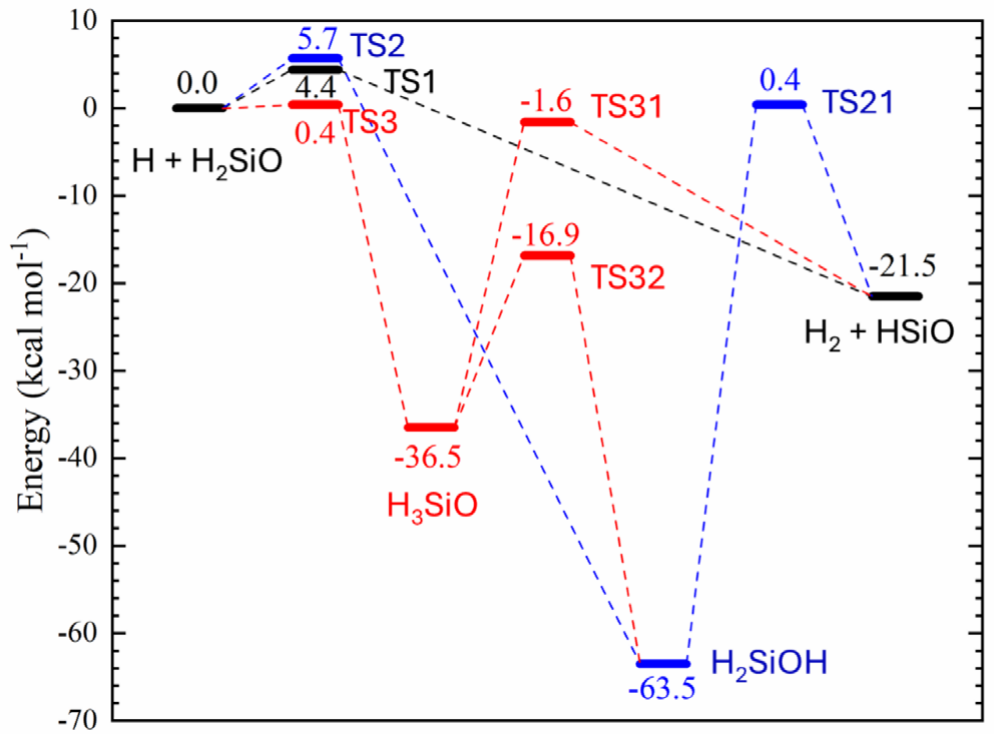
Electronic energy (in kcal mol^−1^) relative to the reactants for reaction paths (R1) (Black), (R2) (Blue), and (R3) (Red) obtained using the ωB97X‐D/aug‐cc‐pVTZ methodology. The figure also shows subsequent reactions from the products of (R2) and (R3) (H_2_SiOH and H_3_SiO) that ultimately can lead to the formation of the (R1) product, that is, molecular hydrogen and the HSiO radical.

The energy diagram for all these stationary states is presented in Figure 2, while the values of the classical barrier height ($V^{‡}$), electronic energy (Δ*E*), and enthalpy of reaction at 0 K (${\Delta H}_{0}^{0}$) for all reactions are provided in Tables S1–S6.

There is no previous accurate experimental thermochemical data available [9] for the reaction energies and enthalpies for these reaction paths, but the ωB97X‐D/aug‐cc‐pVTZ and CCSD(T)/aug‐cc‐pVTZ should provide good estimates. It is also worth mentioning that the ωB97X‐D/aug‐cc‐pVTZ results present a mean absolute deviation (MAD) of 1.4 kcal mol^−1^ when compared to CCSD(T)/aug‐cc‐pVTZ. These values are also in good agreement with CCSD(T)/aug‐cc‐pV5Z single‐point calculations, presenting a MAD of 1.6 kcal mol^−1^. The ωB97X‐D/aug‐cc‐pVTZ and CCSD(T) methods predict values of electronic reaction energies in excellent agreement with the composite methods G2 [39] and G4 [40].

Initially, we compared the energies of the M06‐2X/aug‐cc‐pVTZ and ωB97X‐D/aug‐cc‐pVTZ methods for the reaction path (R1) (Table S1). M06‐2X and ωB97X‐D are density functionals parameterized to describe the kinetics of chemical reactions, but with different emphases in their development. The M06‐2X functional, developed by Zhao and Truhlar [18], is particularly suited for calculating activation barriers in chemical reactions, whereas ωB97X‐D, introduced by Chai and Head‐Gordon [19], is a hybrid functional with long‐range dispersion correction (*D*), designed to describe long‐range interactions and dispersion forces accurately.

Regarding Table S1, the comparison of calculations for the reaction path (R1) shows that the M06‐2X/aug‐cc‐pVTZ method presents the highest classical barrier height (4.7 kcal mol^−1^). In comparison, the CCSD(T)/aug‐cc‐pVTZ shows the lowest value (4.1 kcal mol^−1^), with ωB97X‐D/aug‐cc‐pVTZ providing an intermediate value of 4.5 kcal mol^−1^. The CCSD(T)/aug‐cc‐pV5Z//CCSD(T)/aug‐cc‐pVTZ method slightly increases to 4.3 kcal mol^−1^. The adiabatic barrier height follows a similar trend, ranging from 3.6 kcal mol^−1^ (CCSD(T)/aug‐cc‐pVTZ) to 4.2 kcal mol^−1^ (M06‐2X and ωB97X‐D). The electronic reaction energies are all exothermic, with values ranging from −22.2 kcal mol^−1^ (CCSD(T)/aug‐cc‐pVTZ) to −21.5 kcal mol^−1^ (CCSD(T)/aug‐cc‐pV5Z//CCSD(T)/aug‐cc‐pVTZ), which are close to those obtained by G2 (−21.7 kcal mol^−1^) [39], and G4 (−20.73 kcal mol^−1^) [40], methods, showing good consistency among the more accurate methods. In terms of enthalpy at 298.15 K, coupled‐cluster and G2/G4 methods converge around −19.7 to −20.1 kcal mol^−1^, while M06‐2X and ωB97X‐D estimate slightly higher values, highlighting a slight tendency towards overestimation.

For the reaction path (R2), it is observed that the CCSD(T) method with different basis sets improves the precision of energy values compared to the DFT (ωB97X‐D) method. The classical barrier height ranges from 5.0 to 5.7 kcal/mol^−1^, while the adiabatic barrier height varies between 5.8 and 6.4 kcal/mol^−1^. The electronic reaction energy indicates that the process is strongly exothermic, with values between −60.8 and −63.5 kcal/mol^−1^. The enthalpy of the reaction at 0 K ranges from −54.2 to −57.0 kcal/mol^−1^. Using larger basis sets, such as the aug‐cc‐pV5Z, tends to slightly reduce the exothermicity and refine the values of the energy barriers.

In Table S2, the calculations for the reaction path (R3), where the CCSD(T)/aug‐cc‐pV5Z//CCSD(T)/aug‐cc‐pVTZ method indicates that there is no saddle point in the PES. The electronic reaction energy is −32.3 kcal mol^−1^, and the enthalpy at 0 K is −27.5 kcal mol^−1^. On the other hand, the ωB97X‐D/aug‐cc‐pVTZ method indicates a shallow saddle point with a classical barrier of 0.4 kcal mol^−1^, an adiabatic barrier of 0.7 kcal mol^−1^, an electronic energy of −36.5 kcal mol^−1^, and an enthalpy of −31.9 kcal mol^−1^. These results indicate that while the DFT ωB97X‐D is useful, it tends to overestimate the barriers and exothermicity of the reaction compared to the CCSD(T) method.

The energy diagram in Figure 2 also shows the possible stationary states interconnecting (R2) and (R3) to (R1) and indicates the kinetic behavior for each path and of the reaction system. Taking into consideration the ωB97X‐D/aug‐cc‐pVTZ results, one can see that the very low (or nonexistent) barrier of (R3) combined with the intermediary thermodynamic stability of its product (compared to (R2) and (R1)) indicates that this pathway serves as an alternative to the formation of (R1) and (R2) products. From the (R3) product (H_3_SiO), the adiabatic barrier to form H_2_ and HSiO is equal to 32.9 kcal mol^−1^, almost the same energy released to form H_3_SiO from the reactants. The adiabatic barrier to transfer the hydrogen from the silicon to the oxygen (R32) is even lower (18.2 kcal mol^−1^) and presents a relatively large stabilization, with a reaction enthalpy at 0 K equal to −25.1 kcal mol^−1^.

The (R2) product (H_2_SiOH) is the most stable stationary state within this system (−57.0 kcal mol^−1^) but can dissociate to form H_2_ and HSiO with an adiabatic barrier equal to 60.0 kcal mol^−1^, which is very large and 3.0 kcal mol^−1^ lower than the energy required to dissociate to the reactants.

Overall, these energetic interconnections indicate that the products of (R1) (HSiO and H_2_) and (R2) (H_2_SiOH) should be observed for this reaction system, while the product of (R3) (H_3_SiO) will mostly be transformed into the product of one of these paths and probably is the dominant pathway to form these products at lower temperatures.

The vibrational frequencies and zero‐point vibrational (ZPVE) energy (kcal mol^−1^) of all species are collected in Tables S7–S10. The ωB97X‐D method predicts lower values for the imaginary frequencies (858.7*i* and 1141.4*i* cm^−1^ for TS1 and TS2, respectively) when compared to CCSD(T)/aug‐cc‐pVTZ (1204.2*i* and 1567.5*i* cm^−1^ for TS1 and TS2, respectively), a difference of about 28% which results in a wider barrier at the top of the PES when using the DFT approximation. On the other hand, the real vibrational modes obtained with ωB97X‐D and CCSD(T) are closer to each other, differing on average by less than 5%.

For the reactant H_2_SiO, only two fundamental frequencies have been measured, which are assigned as the stretching Si = O at 1202 cm^−1^ and the bending mode in the *x* *y* plane at 697 cm^−1^ [41]. The harmonic frequencies computed by ωB97X‐D (1259.4 and 682.8 cm^−1^) and coupled‐cluster methods (1192.5 and 676.9 cm^−1^) predict close values for these modes. To the best of the authors' knowledge, there are no vibrational spectroscopy measurements for the HSiO, H_2_SiOH, and H_3_SiO radicals available in the literature.

### 3.2 Chemical Kinetics

The thermal rate constants of the reaction paths (R1) and (R2) were calculated using the VTST‐IOC method, while VRC was used to compute the thermal rate constants of (R3). In the VTST‐IOC approach, the low‐level reaction path (R1) was built with the ωB97X‐D/aug‐cc‐pVTZ. For high‐level corrections, the CCSD(T)/aug‐cc‐pV5Z method was employed to improve the values of electronic barrier height ($V^{‡}$) and reaction energy (Δ*E*), while the benchmark CCSD(T)/aug‐cc‐pVTZ approach was used in the corrections of the vibrational frequencies of the reactants, transition state, and products.

The minimum energy potential (*V*_MEP_) and the adiabatic ground‐state energy ($V_{a}^{G}$) (of R1 and R2) and the free energy of (R3) are given in Figures S1–S3. All curves show smooth behavior along the reaction path around the saddle point, and the electronic structure calculations do not predict the existence of a reactant or product complex for this reaction system.

Tables S11–S13 list the rate constants (cm^3^ molecule^−1^ s^−1^) of ((R1), (R2), (R3)), respectively, calculated in the temperature range of 250–2500 K employing the TST, CVT, CVT/ZCT, and CVT/SCT methods for (R1) and (R2), and VRC/VTST for (R3). In the VTST/MT theory, the variational effect (recrossing effect) is measured as the ratio between CVT (*s* = s*) and TST (*s* = 0, at the saddle point) rate constants. In the temperature range of 250–1000 K, the ratio of *k* ^CVT^/*k* ^TST^ is very different depending on the reaction path. For (R1), the *k* ^CVT^/*k* ^TST^ varies from 0.98 to 0.99 for all temperatures, the maximum of the free‐energy curve shows displacements ($s^{*{\Delta G}_{a}^{v}}$) close to 0.01, indicating no variational effects. On the other hand, for (R2), the k^CVT^/k^TST^ is closer to 0.1 for all temperatures, presenting high displacements ($s^{*{\Delta G}_{a}^{v}}$) ranging from 0.1 (at 250 K) to 0.18 (at 2500 K).

A procedure to estimate the reaction‐path curvature is to compute the skew angle (β) between the entrance and exit valleys of the PES [42, 43]. Garret and Truhlar [43] have compiled collinear three‐atom reactions between the values of the skew angle and the transmission coefficients of hydrogen abstraction reactions of the type A + BC → AB + C, where A, B, and C may be atoms or groups of atoms.

In the study of various MEP, this rule predicts that larger values of the skew angle, that is, close to 90° indicate the predominance of small curvature tunneling, and smaller values closer to ~0° indicate large curve tunneling effects due to the presence of swath paths on the PES [43, 44]. The skew angle is defined by [42, 43],

(4) $$\beta =\arccos{\left(\frac{m_{A}m_{C}}{m_{\mathrm{AB}}m_{\mathrm{BC}}}\right)}^{1/2}$$

Calculations with Equation (4) and considering A (hydrogen), B (hydrogen), and C (Si) give a β value of 46.9° suggesting an intermediate reaction‐path curvature [42] and predominance of small multidimensional tunneling for its chemical kinetics. For comparison, the benchmark hydrogen abstraction reaction Cl + CH_4_ → CH_3_ + HCl [44, 45], whose chemical kinetics are controlled by large‐curvature multidimensional tunneling, has a skew angle equal to 16.3°.

The present calculations of rate constants (Tables S11–S15) confirm this conjecture and show that the predominance of SCT tunneling corrections practically disappears at 1000 K. The CVT, CVT/ZCT, and CVT/SCT rate constants show that all values practically converged at 1500 K for both (R1) and (R2).

**FIGURE 3:**
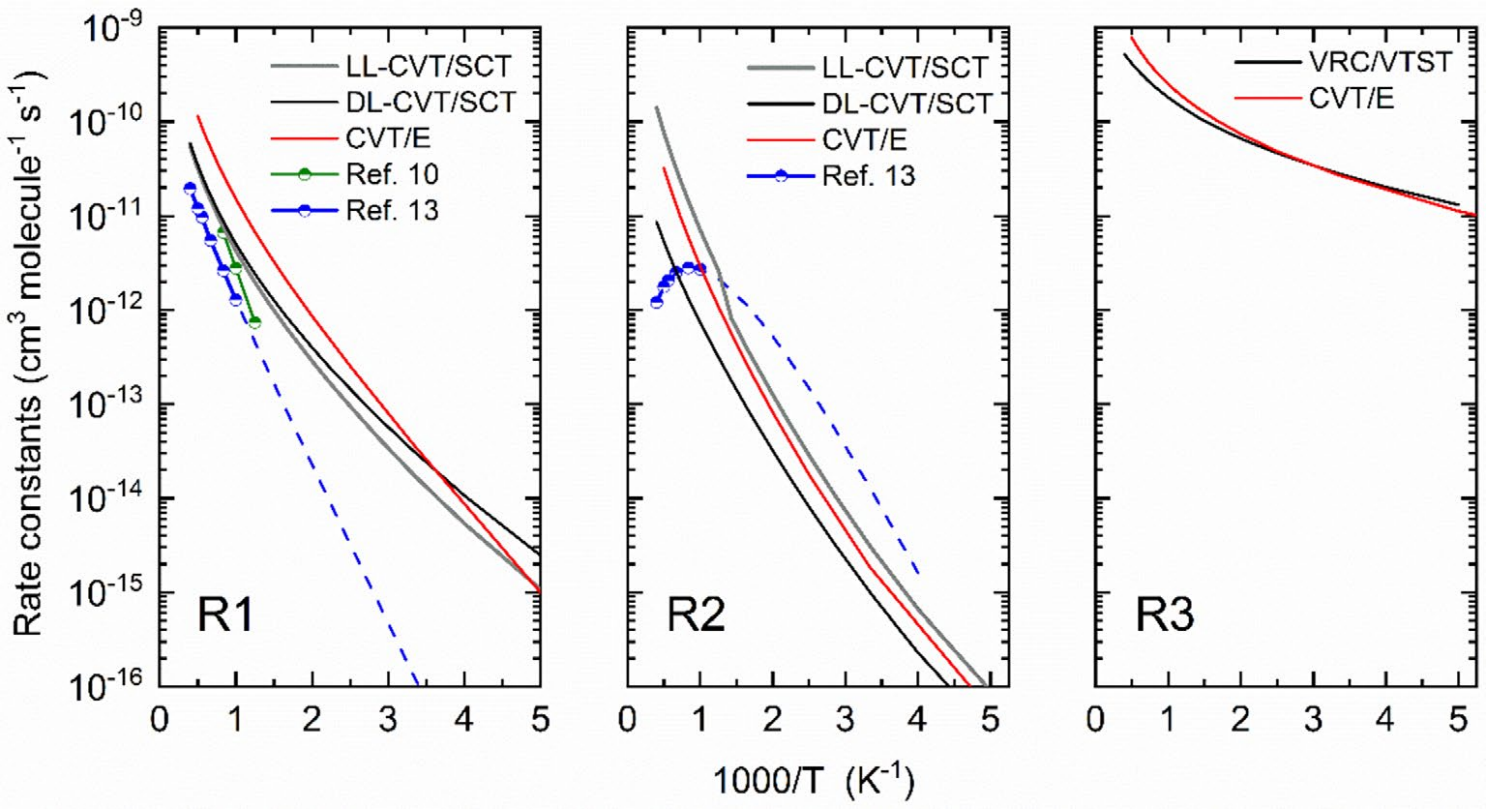
Arrhenius plots of the elementary reactions H + H_2_SiO → H_2_ + HSiO (R1), H + H_2_SiO → H_2_SiOH (R2), and H + H_2_SiO → H_3_SiO (R3). The rate constants were computed by CVT/SCT for paths (R1) and (R2), and VRC/VTST for (R3). Previous estimates from the literature are also included, as well as the high‐pressure limit CVT/Eckart used in the RRKM and EGME calculations.

Figure 3 gives Arrhenius plots of rate constants computed by CVT/SCT for paths (R1) and (R2) and VRC/VTST for (R3). Previous estimates from the literature are also included, as well as the CVT/Eckart used in the subsequent EGME calculations. As discussed above, previous studies of the rate constants of ((R1), (R2), (R3)) were based on empirical correlations of global kinetic reactions involving CH_4_ combustion [11, 12] and BAC and Hartree–Fock electronic structure calculations.

As can be seen in Figure 3, there is a general agreement between the calculated CVT/SCT data (black and gray lines) and the previous literature data for the high‐temperature rate constants for (R1) (blue and green solid lines), as expected since these data were calibrated or derived from experimental data at these temperature ranges. At lower temperatures, the calculations indicate a much higher rate constants than those expected from extrapolations of the literature data to lower temperatures (dashed blue line). For (R2), shown in Figure 3, one can see a similar pattern, with a numerical agreement between the calculated and previous literature data, although there is a qualitative discrepancy in the temperature dependency at higher temperatures. However, this “discrepancy” is artificial since the values obtained by Zachariah and Tsang [13] represent the phenomenological rate constant, that is, they consider the decomposition of the H_2_SiOH intermediary at higher temperatures, as is discussed later in the present paper. The rate constants of (R3) (Figure 3) presented very high values with low dependency on temperature, as expected given that the barrier height at the ωB97X‐D/aug‐cc‐pVTZ level is about 0.4 kcal mol^−1^. This indicates a prompt availability of H_3_SiO in the reaction system, which can decompose to the other products, especially H_2_SiOH, as discussed in the energetics section. The CVT/Eckart rate constants also compare well with the CVT/SCT values, despite not including explicit information about the PES around the saddle point, only the imaginary frequency.

The thermal rate constants shown in Figure 3 were fitted to an Arrhenius expression with four parameters [46], and the fitted parameters for all reaction paths are provided in Table S16, Alongside the activation energy derived from this fitting (Table S17 and Figure S4).

**FIGURE 4:**
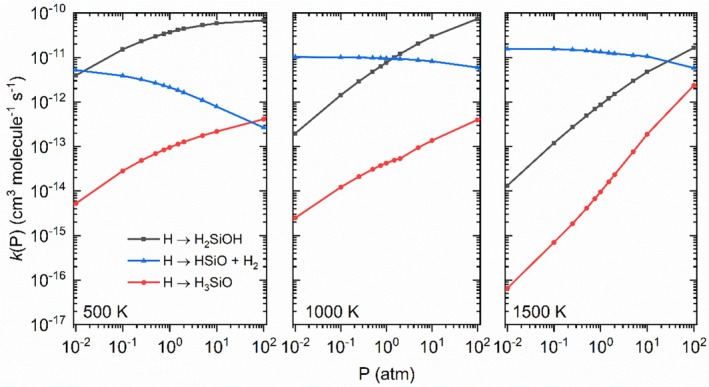
Bartis–Widom phenomenological rate coefficients obtained with the EGME approach for the reaction system H + H_2_SiO (excess reactant).

The Bartis−Widom phenomenological rate coefficients [47, 48] obtained from the EGME approach were calculated for the formation of the products, providing an apparent rate constant for ((R1), (R2), (R3)). The pressure dependence of these coefficients is shown in Figure 4 for three different temperatures (500, 1000, and 1500 K), and the complete data is provided in Table S18.

The phenomenological rate constants in Figure 4 show a competition between the formation of H_2_SiOH and the bimolecular products over a wide range of temperatures and pressures, while the formation of H_3_SiO is in most scenarios one to two orders of magnitude lower. As the temperature increases, the trends for the phenomenological rate constants of H_2_SiOH and the bimolecular products are inverted and the coefficient for the formation of H_2_SiOH lowers, in agreement with the previous literature data [13]. Also, the formation of the unimolecular species presents higher dependence on the pressure, especially at higher temperatures, and its behavior is qualitatively different from the bimolecular reaction, as expected.

**FIGURE 5:**
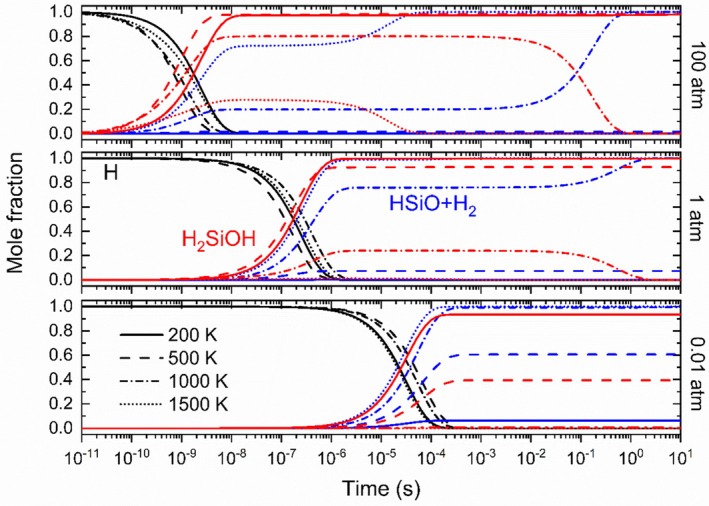
Time evolution of the species for several temperatures (different line styles) and pressures, 0.01 atm (bottom), 1 atm (center), and 100 atm (top). Hydrogen is shown in black, H_2_SiOH in red, and HSiO + H_2_ in blue.

The time evolution of the species is shown in Figure 5, considering various temperatures, from 200 to 1500 K, at 0.01, 1, and 100 atm. As expected from the energy diagram, the time‐dependent species profiles confirm that for lower temperatures (up to 500 K) and high pressures, the reaction system advances only up to the formation of the intermediate H_2_SiOH, which is mostly produced through the (R3) → (R3‐2) sequence. At higher temperatures, the formation of HSiO + H_2_ is favored, formed both directly through (R1) and from the decomposition of the intermediates (R2‐1) and (R3‐1). After the consumption of the deficient reactant (around 3 μs at 1 atm), the fraction of the intermediate H_2_SiOH is around 92% at 500 K, but only 24% at 1000 K, and is slowly decomposed to HSiO + H_2_. At 1500 K, the molar fraction of this intermediate does not surpass 1.5% and is much shorter‐ lived.

Since the analysis of the KIE has significant implications for the understanding of reaction mechanisms and provides information on the structure and stability of transition states, we also calculated the KIE for hydrogen production at high temperatures, in which (R1) dominates. In this context, two reactions were considered, one deuterating the hydrogen radical (R1A) and the other deuterating the hydrogens from the oxosilane (R1B),

(R1A) $$D+H_{2}\mathrm{SiO}\to \text{HSiO}+\mathrm{DH}$$

(R1B) $$H+D_{2}\mathrm{SiO}\to \text{DSiO}+\mathrm{DH}$$

**FIGURE 6:**
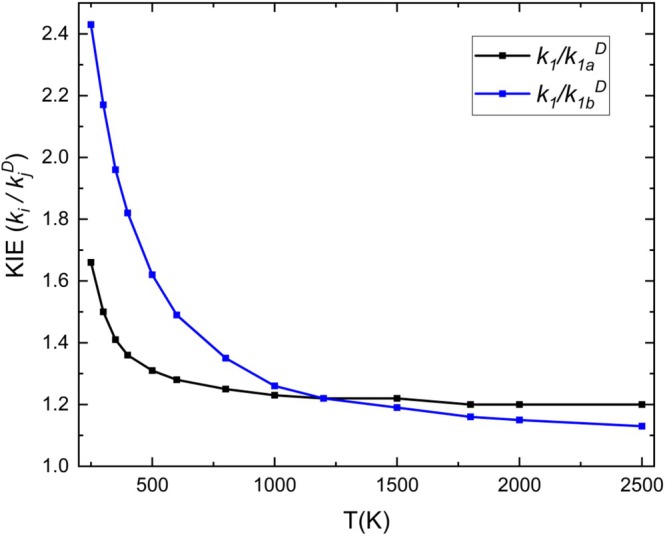
Values of *k*_*1*_^*H*^ */k*_*1A*_^*D*^ and *k*_*1*_^*H*^ */k*_*1B*_^*D*^ vs. *T* (K) obtained with CVT/SCT rate constants.

The KIEs of reactions (R1A) and (R1B) calculated at the CVT/SCT level, given by the ratios of the hydrogen and deuterated reaction rate constants, (*k*_*1*_^*H*^ */k*_*1A*_^*D*^) and (*k*_*1*_^*H*^ */k*_*1B*_^*D*^), are shown in Figure 6. The KIE calculated at CVT, CVT/ZCT, and CVT/SCT levels is given in Table S19. To the best of our knowledge, no experimental isotope data are available for comparison.

The values of the KIEs represent the balance between the curvature of the reactive path and the variation of zero‐point vibration energy [49]. The rate constants of (*k*_1b_^D^) (H + D_2_SiO) present larger variational effects, varying from 2.43 (250 K) to 1.19 (1500 K), while the KIE of (*k*_1a_^D^) (D + H_2_SiO) varies much less, from 1.66 (250 K) to 1.22 (1500 K). This behavior is mostly due to two underlying effects. On the one hand, there is lower tunneling in the deuterated reactions; the deuterium transition mode of (R1A) reduces from 1204*i* to 982*i* cm^−1^, reflecting a wider surface at the top of the PES and therefore, a smaller tunneling coefficient. This effect is attenuated for (R1B), in which the deuterium transition mode reduces from 1204*i* to 1045*i* cm^−1^. On the other hand, there is the zero‐point effect (ZPE) on the vibrationally adiabatic surface; the ZPE of (R1) lowers the adiabatic barrier height by 0.38 kcal mol^−1^ when compared to the classical barrier height (at ωB97XD/aug‐cc‐pVTZ level). For the deuterated reactions, the barrier lowers by 0.58 kcal mol^−1^ for (R1A) and only 0.05 kcal mol^−1^ for (R1B) when including the ZPE. This counteracts the effect of lower tunneling for (R1A) and makes it closer to (R1) at lower temperatures and delays the asymptotic effect of KIE to higher temperatures. In comparison, (R1B) presents reduced rates from both tunneling and ZPE, increasing the KIE at lower temperatures and increasing the decay to the asymptotic limit at higher temperatures.

## 4 Conclusions

The ωB97X‐D and CCSD(T) methods, along with a series of basis sets, were employed to characterize the stationary states (electronic energies, equilibrium geometries, and harmonic vibrational frequencies) of the H + H_2_SiO reaction system forming molecular hydrogen and HSiO (R1), H_2_SiOH (R2), or H_3_SiO (R3). Best estimate calculations were computed using the CCSD(T)/aug‐cc‐pV5Z//CCSD(T)/aug‐cc‐pVTZ method. The ωB97X‐D/aug‐cc‐pVTZ and CCSD(T) methods using the aug‐cc‐pVTZ basis set predict electronic surfaces in good agreement, with a MAD of only 1.4 kcal mol^−1^. The RP‐VTST/MT and VRC‐TST rate constants are computed assuming high‐pressure conditions, and low‐level electronic structure calculations were carried out with a ωB97X‐D/aug‐cc‐pVTZ low‐level surface. The CCSD(T)/aug‐cc‐pVTZ methodology is employed in the calculations of vibrational frequencies of reactants, transition state, and products.

The reaction paths (R1) and (R2) have a small skew angle (46.9°) from which intermediate tunneling effects would be expected. This prediction is confirmed in the VTST/MT calculations, which demonstrate the importance of including SCT tunneling corrections for the determination of rate constants from low (250 K) to intermediate temperature values (~1000 K). The reaction path (R3) is barrierless and presents high reaction rates that are dominant at low temperatures. EGME time‐dependent species profiles showed that for lower temperatures (up to 500 K), the main reaction product is H_2_SiOH (through (R3) followed by (R3‐2)), while at higher temperatures, the formation of HSiO + H_2_ is favored. Bartis−Widom phenomenological rate coefficients were also provided for the formation of the products (H_3_SiO, H_2_SiOH, and HSiO + H_2_). To the best of our knowledge, there are no accurate calculations of rate constants in the range 250–2500 K available in the literature; therefore, this study provides important data for the modeling of the global kinetics of silane oxidation.

The KIEs as a function of the temperature are computed for the combination of H + H_2_SiO (R1) and the deuterated species D + SiH_2_O (R1A) and H + SiD_2_O (R1B). Values of CVT/SCT of both *k*_1_^H^/*k*_1a_^D^ (R1/R1A) and *k*_1_^H^ */k*_1b_^D^ (R1/R1B) show normal KIE (≥ 1.0). Calculated KIEs are relatively small, dominated by a balance between the zero‐point vibrational energies and SCT tunneling effects.

## Supporting information


**Table S1.** Barrier height, reaction energy, and enthalpy of reaction (${\mathrm{\Delta H}}_{T}^{0}$) (in kcal mol^−1^) of the H + H_2_SiO → HSiO + H_2_ reaction (R1).
**Table S2.** Barrier height, reaction energy, and enthalpy of reaction (${\mathrm{\Delta H}}_{0}^{0}$) (in kcal mol^−1^) of the H + H_2_SiO → H_2_SiOH reaction (R2).
**Table S3.** Barrier height, reaction energy, and enthalpy of reaction (${\mathrm{\Delta H}}_{0}^{0}$) (in kcal mol^−1^) of the H + H_2_SiO → H_3_SiOH reaction (R3).
**Table S4.** Barrier height, reaction energy, and enthalpy of reaction (${\mathrm{\Delta H}}_{T}^{0}$) (in kcal mol^−1^) of the H_2_SiOH → HSiO + H_2_ reaction (R21).
**Table S5.** Barrier height, reaction energy, and enthalpy of reaction (${\mathrm{\Delta H}}_{T}^{0}$) (in kcal mol^−1^) of the H_3_SiO → HSiO + H_2_ reaction (R31).
**Table S6.** Barrier height, reaction energy, and enthalpy of reaction (${\mathrm{\Delta H}}_{T}^{0}$) (in kcal mol^−1^) of the H_3_SiO → H_2_SiOH reaction (R32).
**Table S7.** Harmonic vibrational frequencies (cm^−1^) and zero‐point vibrational energy (ZPVE) (in kcal mol^−1^) of the reactants and products of all reaction paths H_2_SiO, HSiO, H_2_, H_2_SiOH, and H_3_SiO calculated with the M06‐2X, ωB97X‐D, CCSD(T) methods, and with the aug‐cc‐pVTZ.
**Table S8.** Harmonic vibrational frequencies (cm^−1^) and zero‐point vibrational energy (ZPVE) (in kcal mol^−1^) of the saddle points of all reaction paths (TS1, TS2, and TS3) calculated with the M06‐2X, ωB97X‐D, CCSD(T) methods with the aug‐cc‐pVTZ.
**Table S9.** ωB97‐XD/aug‐cc‐pVTZ vibrational harmonic frequencies (cm^−1^) and zero‐point vibrational energy (ZPVE) (kcal mol^−1^) for H_2_, HSiO, H_2_SiO, and transition state and the deuterated species of R1.
**Table S10.** CCSD(T)/aug‐cc‐pVTZ vibrational harmonic frequencies (cm^−1^) and zero‐point vibrational energy (ZPVE) (kcal mol^−1^) for H_2_, HSiO, H_2_SiO, HSiO, and transition state and the deuterated species.
**Figure S1.** Electronic energy potential (V_MEP_) adiabatic ground‐state energy ($V_{a}^{G}$) curves along the mass‐scaled reaction coordinate (s) for (R1).
**Figure S2.** Electronic energy potential (V_MEP_) adiabatic ground‐state energy ($V_{a}^{G}$) curves along the mass‐scaled reaction coordinate (s) for (R2).
**Figure S3.** Free energy curves at the lowest and highest temperatures considered along the mass‐scaled reaction coordinate (s) for (R3).
**Table S11.** Rate constants for R1: H + SiH_2_O → HSiO + H_2_ (in cm^3^ molecule s^−1^).
**Table S12.** Rate constants for R2: H + SiH_2_O → H_2_SiOH (in cm^3^ molecule s^−1^).
**Table S13.** Rate constants for R3: H + SiH_2_O → H_3_SiO (in cm^3^ molecule s^−1^). The rate constant values refer only to the low‐level electronic structure calculation (ωB97X‐D/aug‐cc‐pVTZ).
**Table S14.** Rate constants for R31: H + SiH_2_O → H_3_SiO (reactant well) → H_2_ + HSiO (in cm^3^ molecule s^−1^). The rate constant values refer only to the low‐level electronic structure calculation (ωB97X‐D/aug‐cc‐pVTZ).
**Table S15.** Rate constants for R32: H_3_SiO → H_2_SiOH (in s^−1^). The rate constant values refer only to the low‐level electronic structure calculation (ωB97X‐D/aug‐cc‐pVTZ).
**Table S16.** Fitted parameters for all the reaction paths studied using a 4‐parameter Arrhenius fitting and the Dual level CVT/SCT, except for R3, which was obtained using VRC/*E,J*‐μVT, and the low‐level surface.
**Table S17.** Activation energy (cal/mol) for all studied paths.
**Figure S4.** Activation energy (cal/mol) for all studied paths.
**Table S18.** Bartis‐Widom Phenomenological Rate Coefficients (in cm^3^ molecule^−1^ s^−1^).
**Table S19.** Kinetic isotope effect of R1/R1A (k_1_^H^/*k*_1a_^D^) and R1/R1B (k_1_^H^/k_1b_^D^) paths.

## Data Availability

The data that support the findings of this study are available in the Supporting Information S1 of this article.
