# Supplementary material for: Rate Constants and Energetics of the H2SiO + H Reaction System: RP‐VTST/MT and VRC Calculations
Source: J Comput Chem. 2025 May 19;46(14):e70115. doi: 10.1002/jcc.70115 (PMC12087265; doi:10.1002/jcc.70115)
Supplement: Supplementary file 1 — Table S1. Barrier height, reaction energy, and enthalpy of reaction (ΔHT0) (in kcal mol−1) of the H + H2SiO → HSiO + H2 reaction (R1). Table S2. Barrier height, reaction energy, and enthalpy of reaction (ΔH00) (in kcal mol−1) of the H + H2SiO → H2SiOH reaction (R2). Table S3. Barrier height, reaction energy, and enthalpy of reaction (ΔH00) (in kcal mol−1) of the H + H2SiO → H3SiOH reaction (R3). Table S4. Barrier height, reaction energy, and enthalpy of reaction (ΔHT0) (in kcal mol−1) of the H2SiOH → HSiO + H2 reaction (R21). Table S5. Barrier height, reaction energy, and enthalpy of reaction (ΔHT0) (in kcal mol−1) of the H3SiO → HSiO + H2 reaction (R31). Table S6. Barrier height, reaction energy, and enthalpy of reaction (ΔHT0) (in kcal mol−1) of the H3SiO → H2SiOH reaction (R32). Table S7. Harmonic vibrational frequencies (cm−1) and zero‐point vibrational energy (ZPVE) (in kcal mol−1) of the reactants and products of all reaction paths H2SiO, HSiO, H2, H2SiOH, and H3SiO calculated with the M06‐2X, ωB97X‐D, CCSD(T) methods, and with the aug‐cc‐pVTZ. Table S8. Harmonic vibrational frequencies (cm−1) and zero‐point vibrational energy (ZPVE) (in kcal mol−1) of the saddle points of all reaction paths (TS1, TS2, and TS3) calculated with the M06‐2X, ωB97X‐D, CCSD(T) methods with the aug‐cc‐pVTZ. Table S9. ωB97‐XD/aug‐cc‐pVTZ vibrational harmonic frequencies (cm−1) and zero‐point vibrational energy (ZPVE) (kcal mol−1) for H2, HSiO, H2SiO, and transition state and the deuterated species of R1. Table S10. CCSD(T)/aug‐cc‐pVTZ vibrational harmonic frequencies (cm−1) and zero‐point vibrational energy (ZPVE) (kcal mol−1) for H2, HSiO, H2SiO, HSiO, and transition state and the deuterated species. Figure S1. Electronic energy potential (VMEP) adiabatic ground‐state energy (VaG) curves along the mass‐scaled reaction coordinate (s) for (R1). Figure S2. Electronic energy potential (VMEP) adiabatic ground‐state energy (VaG) curves along the mass‐scaled reaction coordinate (s) for (R2 [file JCC-46-0-s001.docx]

**Rate Constants and Energetics of the H_2_SiO + H Reaction System: RP-VTST/MT and VRC calculations**

Marcelo André Petry Pontes ^1,2,3^, Edson Firmino Viana de Carvalho ^4*^, Luiz Fernando de Araújo Ferrão ^1,2*^, Francisco Bolivar Correto Machado^1,2*^, and Orlando Roberto-Neto ^5*^

*^1^Departamento de Química, Instituto Tecnológico da Aeronáutica, 12228-900, São José dos Campos, São Paulo, Brazil*

*^2^Laboratório de Computação Científica Avançada e Modelamento (Lab – CCAM), Instituto Tecnológico da Aeronáutica, 12228-900, José dos Campos, São Paulo, Brazil.*

*^3^Departamento de Engenharia, Faculdade Anhanguera de Jacareí, 12307-130, Jacareí, São Paulo, Brazil*

*^4^Departamento de Física, Universidade Federal do Maranhão, 65085-580, São Luís, Maranhão, Brazil*

*^5^Divisão de Aerotermodinâmica e Hipersônica, Instituto de Estudos Avançados, São José dos Campos, 12228-001, São Paulo, Brazil*

**Supplementary Material**

[Table S1 2](#_Toc195100786)

[Table S2 2](#_Toc195100787)

[Table S3 2](#_Toc195100788)

[Table S4 3](#_Toc195100789)

[Table S5 3](#_Toc195100790)

[Table S6 3](#_Toc195100791)

[Table S7 4](#_Toc195100792)

[Table S8 5](#_Toc195100793)

[Table S9 6](#_Toc195100794)

[Table S10 8](#_Toc195100795)

[Figure S1 9](#_Toc195100796)

[Figure S2 10](#_Toc195100797)

[Figure S3 11](#_Toc195100798)

[Table S11 12](#_Toc195100799)

[Table S12 13](#_Toc195100800)

[Table S13 14](#_Toc195100801)

[Table S14 15](#_Toc195100802)

[Table S15 16](#_Toc195100803)

[Table S16 17](#_Toc195100804)

[Table S17 18](#_Toc195100805)

[Figure S4 19](#_Toc195100806)

[Table S18 20](#_Toc195100807)

[Table S19 21](#_Toc195100808)

[Cartesian coordinates 22](#_Toc195100809)

Table S1 Barrier height, reaction energy and enthalpy of reaction (${\Delta H}_{T}^{0}$) (in kcal.mol^−1^) of the H + H_2_SiO → HSiO + H_2_ reaction (R1)

| Method | $V^{\ddagger}$ | $\Delta V_{a,V}^{\ddagger}$ | ΔE | ${\Delta H}_{0}^{0}$ |
| --- | --- | --- | --- | --- |
| M06-2X/aug-cc-pVTZ | 4.74 | 4.16 | −22.13 | −21.94 |
| ωB97X-D/aug-cc-pVTZ | 4.53 | 4.15 | −21.53 | −21.29 |
| CCSD(T)/aug-cc-pVTZ | 4.14 | 3.42 | −22.15 | −22.08 |
| CCSD(T)/aug-cc-pV5Z^a^ | 4.41 | 3.69 | −21.23 | -21.16 |
| G2 [38] |  |  | −21.70 | −22.42 |
| G4 [39] |  |  | −20.73 | −22.14 |

^a^CCSD(T)/aug-cc-pVTZ geometries and frequencies

Table S2 Barrier height, reaction energy and enthalpy of reaction (${\Delta H}_{0}^{0}$) (in kcal.mol^−1^) of the H + H_2_SiO → H_2_SiOH reaction (R2)

| Method | $V^{\ddagger}$ | $\Delta V_{a,V}^{\ddagger}$ | ΔE | ${\Delta H}_{0}^{0}$ |
| --- | --- | --- | --- | --- |
| ωB97X-D/aug-cc-pVTZ | 5.7 | 6.4 | −63.5 | −57.0 |
| CCSD(T)/aug-cc-pVTZ | 5.0 | 5.8 | −61.8 | −55.2 |
| CCSD(T)/aug-cc-pV5Z^a^ | 5.2 | 6.0 | −60.8 | −54.2 |

^a^CCSD(T)/aug-cc-pVTZ geometries and frequencies.

Table S3 Barrier height, reaction energy and enthalpy of reaction (${\Delta H}_{0}^{0}$) (in kcal.mol^−1^) of the H + H_2_SiO → H_3_SiOH reaction (R3)

| Method | $V^{\ddagger}$ | $\Delta V_{a,V}^{\ddagger}$ | ΔE | ${\Delta H}_{0}^{0}$ |
| --- | --- | --- | --- | --- |
| ωB97X-D/aug-cc-pVTZ | 0.4 | 0.7 | −36.5 | −31.9 |
| CCSD(T)/aug-cc-pVTZ | −−− | −−− | −33.5 | −28.7 |
| CCSD(T)/aug-cc-pV5Z^a^ | −−− | −−− | −32.3 | −27.5 |

^a^CCSD(T)/aug-cc-pVTZ geometries and frequencies.

Table S4 Barrier height, reaction energy and enthalpy of reaction (${\Delta H}_{T}^{0}$) (in kcal.mol^−1^) of the H_2_SiOH → HSiO + H_2_ reaction (R21)

| Method | $V^{\ddagger}$ | $\Delta V_{a,V}^{\ddagger}$ | ΔE | ${\Delta H}_{0}^{0}$ |
| --- | --- | --- | --- | --- |
| ωB97X-D/aug-cc-pVTZ | 63.9 | 60.6 | 42.0 | 35.7 |

Table S5 Barrier height, reaction energy and enthalpy of reaction (${\Delta H}_{T}^{0}$) (in kcal.mol^−1^) of the H_3_SiO → HSiO + H_2_ reaction (R31)

| Method | $V^{\ddagger}$ | $\Delta V_{a,V}^{\ddagger}$ | ΔE | ${\Delta H}_{0}^{0}$ |
| --- | --- | --- | --- | --- |
| ωB97X-D/aug-cc-pVTZ | 34.9 | 32.9 | 15.0 | 10.6 |

Table S6 Barrier height, reaction energy and enthalpy of reaction (${\Delta H}_{T}^{0}$) (in kcal.mol^−1^) of the H_3_SiO → H_2_SiOH reaction (R32)

| Method | $V^{\ddagger}$ | $\Delta V_{a,V}^{\ddagger}$ | ΔE | ${\Delta H}_{0}^{0}$ |
| --- | --- | --- | --- | --- |
| ωB97X-D/aug-cc-pVTZ | 19.7 | 18.2 | -27.0 | -25.1 |

Table S7. Harmonic vibrational frequencies (cm^−1^) and zero-point vibrational energy (ZPVE) (in kcal.mol^−1^) of the reactants and products of all reaction paths H_2_SiO, HSiO, H_2_, H_2_SiOH, and H_3_SiO calculated with the M06-2X, ωB97X-D, CCSD(T) methods and with the aug-cc-pVTZ.

|  |  | M06-2X | ωB97X-D | CCSD(T) | Exp.: |
| --- | --- | --- | --- | --- | --- |
|  | ν_1_ | 2250.3 | 2205.1 | 2261.2 |  |
| H_2_SiO | ν_2_ | 2236.6 | 2196.7 | 2243.4 |  |
|  | ν_3_ | 1271.98 | 1259.38 | 1192.5 | 1202^a^ |
|  | ν_4_ | 1011.3 | 1002.9 | 997.3 |  |
|  | ν_5_ | 720.8 | 712.81 | 695.9 |  |
|  | ν_6_ | 689.0 | 682.8 | 676.9 | 697^a^ |
|  | *ZPVE* | *11.7* | *11.5* | *11.5* |  |
|  |  |  |  |  |  |
|  | ν_1_ | 2068.0 | 1951.6 | 1894.4 |  |
| HSiO | ν_2_ | 1240.5 | 1210.6 | 1165.4 |  |
|  | ν_3_ | 680.7 | 629.6 | 654.5 |  |
|  | *ZPVE* | *5.7* | *5.4* | *5.3* |  |
|  |  |  |  |  |  |
| H_2_ | ν_1_ | 4465.6 | 4434.1 | 4401.1 | 4401.2^b^ |
|  | *ZPVE* | *6.38* | *6.34* | *6.29* |  |
|  |  |  |  |  |  |
|  | υ_1_ |  | 3950.9 | 3886.0 |  |
|  | υ_2_ |  | 2170.9 | 2233.0 |  |
|  | υ_3_ |  | 2114.9 | 2168.3 |  |
| H_2_SiOH | υ_4_ |  | 928.3 | 925.6 |  |
|  | υ_5_ |  | 898.7 | 900.3 |  |
|  | υ_6_ |  | 853.1 | 844.4 |  |
|  | υ_7_ |  | 775.0 | 777.2 |  |
|  | υ_8_ |  | 677.3 | 675.9 |  |
|  | υ_9_ |  | 273.0 | 259.6 |  |
|  | *ZPVE* |  | 18.1 | 18.1 |  |
|  |  |  |  |  |  |
|  | υ_1_ |  | 2203.5 | 2252.3 |  |
|  | υ_2_ |  | 2201.4 | 2247.1 |  |
|  | υ_3_ |  | 2167.4 | 2221.8 |  |
|  | υ_4_ |  | 990.0 | 985.2 |  |
| H_3_SiO | υ_5_ |  | 914.1 | 930.0 |  |
|  | υ_6_ |  | 900.8 | 910.6 |  |
|  | υ_7_ |  | 827.6 | 824.7 |  |
|  | υ_8_ |  | 557.0 | 573.9 |  |
|  | υ_9_ |  | 547.4 | 485.1 |  |
|  | *ZPVE* |  | 16.2 | 16.3 |  |

^a^[36]

^b^[37]

Table S8 Harmonic vibrational frequencies (cm^−1^) and zero-point vibrational energy (ZPVE) (in kcal.mol^−1^) of the saddle points of all reaction paths (TS1, TS2, and TS3) calculated with the M06-2X, ωB97X-D, CCSD(T) methods with the aug-cc-pVTZ.

|  | TS1 | | | | TS2 | | TS3 | |
| --- | --- | --- | --- | --- | --- | --- | --- | --- |
| Mode | M06-2X | ωB97X-D | CCSD(T) | Ref. [13]^a^ | ωB97X-D | CCSD(T) | ωB97X-D | CCSD(T) |
| υ_1_ | 2221.1 | 2162.6 | 2201.9 | 2081.9 | 2240.0 | 2270.8 | 2217.2 | - |
| υ_2_ | 1403.5 | 1514.7 | 1295.6 | 1208.2 | 2222.7 | 2246.1 | 2210.0 | - |
| υ_3_ | 1258.2 | 1248.6 | 1170.0 | 1048.5 | 1159.4 | 1166.4 | 1259.0 | - |
| υ_4_ | 992.3 | 995.4 | 992.3 | 974.5 | 987.4 | 981.9 | 1002.1 | - |
| υ_5_ | 787.8 | 763.1 | 774.9 | 916.3 | 687.1 | 703.3 | 715.4 | - |
| υ_6_ | 697.4 | 692.4 | 691.6 | 747.1 | 679.4 | 670.5 | 685.3 | - |
| υ_7_ | 258.9 | 247.4 | 258.3 | 332.9 | 339.7 | 374.8 | 155.2 | - |
| υ_8_ | 158.4 | 168.3 | 179.0 | 222.7 | 196.5 | 200.1 | 86.9 | - |
| υ_9_ | *994.9i* | *858.7i* | *1204.2i* | 1927.1i | 1141.4i | 1567.5i | 175.8i | - |
| ZPVE | 11.1 | 11.1 | 10.8 |  | 12.2 | 12.3 | 11.9 | - |

^a^HF/6-31G(d) corrected by a scale factor of 0.89.

Table S9 ωB97-XD/aug-cc-pVTZ vibrational harmonic frequencies (cm^−1^) and zero-point vibrational energy (ZPVE) (kcal.mol^−1^) for H_2_, HSiO. H_2_SiO, and transition state and the deuterated species of R1.

| Mode |  |  |  |  | ωB97-XD |  | TS1 | TS1A | TS1B |
| --- | --- | --- | --- | --- | --- | --- | --- | --- | --- |
|  | H_2_ | DH | HSiO | DSiO | SiH_2_O | SiD_2_O | HSiH_2_O | DSiH_2_O | HD_2_SiO |
| υ_1_ | 4434.05 | 3840.42 | 1964.21 | 1417.16 | 2205.06 | 1596.60 | 2162.65 | 2157.57 | 1559.14 |
| υ_2_ |  |  | 1211.42 | 1206.10 | 2196.65 | 1581.94 | 1514.76 | 1448.02 | 1279.86 |
| υ_3_ |  |  | 627.26 | 472.86 | 1259.38 | 1243.14 | 1248.60 | 1244.16 | 1132.52 |
| υ_4_ |  |  |  |  | 1002.97 | 721.29 | 995.43 | 992.77 | 718.16 |
| υ_5_ |  |  |  |  | 712.81 | 546.51 | 763.19 | 760.92 | 579.08 |
| υ_6_ |  |  |  |  | 682.87 | 532.24 | 692.44 | 689.39 | 532.35 |
| υ_7_ |  |  |  |  |  |  | 247.43 | 233.85 | 215.31 |
| υ_8_ |  |  |  |  |  |  | 168.32 | 131.88 | 164.99 |
| υ_9_ |  |  |  |  |  |  | *858.71i* | 651.90i | *802.37i* |
| *ZPVE* | *6.33* | *5.49* | *5.42* | *4.43* | *11.52* | *8.89* | *11.14* | 10.94 | *8.84* |

Table S10 CCSD(T)/aug-cc-pVTZ vibrational harmonic frequencies (cm^−1^) and zero-point vibrational energy (ZPVE) (kcal.mol^−1^) for H_2_, HSiO. H_2_SiO, HSiO, and transition state and the deuterated species.

| Mode |  |  |  |  |  |  |  | TS1 | TS1A | TS1B |
| --- | --- | --- | --- | --- | --- | --- | --- | --- | --- | --- |
|  | H_2_ | DH | HSiO | DSiO |  | H_2_SiO | SiD_2_O | HSiH_2_O | DSiH_2_O | HD_2_SiO |
| υ_1_ | 4401.10 | 3811.94 | 1894.38 | 1366.91 |  | 2261.23 | 1637.55 | 2201.89 | 2201.12 | 1589.05 |
| υ_2_ |  |  | 1165.41 | 1160.44 |  | 2243.43 | 1612.31 | 1295.62 | 1221.45 | 1203.72 |
| υ_3_ |  |  | 654.45 | 491.38 |  | 1192.47 | 1178.49 | 1169.99 | 1104.49 | 1029.71 |
| υ_4_ |  |  |  |  |  | 997.33 | 718.160 | 992.30 | 979.11 | 719.55 |
| υ_5_ |  |  |  |  |  | 695.88 | 533.35 | 774.85 | 765.66 | 585.71 |
| υ_6_ |  |  |  |  |  | 676.90 | 526.14 | 691.63 | 683.35 | 528.61 |
| υ_7_ |  |  |  |  |  |  |  | 258.34 | 233.17 | 216.49 |
| υ_8_ |  |  |  |  |  |  |  | 178.98 | 136.13 | 172.22 |
| υ_9_ |  |  |  |  |  |  |  | *1204.21i* | *982.15i* | *1045.06i* |
| *ZPVE* | *6.29* | *5.45* | *5.31* | *4.32* |  | *11.53* | *8.88* | *10.81* | *10.47* | *8.64* |





Figure S1 Electronic energy potential energy potential (V_MEP_) adiabatic ground-state energy ($V_{a}^{G}$) curves along the mass-scaled reaction coordinate (s) for (R1).





Figure S2 Electronic energy potential energy potential (V_MEP_) adiabatic ground-state energy ($V_{a}^{G}$) curves along the mass-scaled reaction coordinate (s) for (R2).





Figure S3 Free energy curves at the lowest and highest temperatures considered along the mass-scaled reaction coordinate (s) for (R3).

Table S11 Rate constants for R1: H + SiH_2_O → HSiO + H_2_ (in cm^3^molecule s^−1^)

| T(K) | TST | CVT | CVT/ZCT | CVT/SCT | Ref.^10,11,12^ | Ref.^13,14**^ | T(K) | CVT/E* |
| --- | --- | --- | --- | --- | --- | --- | --- | --- |
| 250 | 5.51E-15 | 4.10E-15 | 7.28E-15 | 1.07E-14 |  | 1.04E-17 | 200 | 9.88E-16 |
| 300 | 2.07E-14 | 1.65E-14 | 2.42E-14 | 3.17E-14 |  | 1.31E-16 | 300 | 3.69E-14 |
| 350 | 5.51E-14 | 4.58E-14 | 5.99E-14 | 7.34E-14 |  | 8.11E-16 | 400 | 2.52E-13 |
| 400 | 1.18E-13 | 1.02E-13 | 1.23E-13 | 1.45E-13 |  | 3.22E-15 | 500 | 8.62E-13 |
| 500 | 3.60E-13 | 3.23E-13 | 3.60E-13 | 3.99E-13 |  | 2.26E-14 | 600 | 2.06E-12 |
| 600 | 8.03E-13 | 7.37E-13 | 7.85E-13 | 8.43E-13 |  | 8.46E-14 | 700 | 4.00E-12 |
| 800 | 2.43E-12 | 2.28E-12 | 2.33E-12 | 2.43E-12 | 7.4E-13 | 4.56E-13 | 800 | 6.79E-12 |
| 1000 | 5.18E-12 | 4.91E-12 | 4.94E-12 | 5.07E-12 | 2.8E-12 | 1.29E-12 | 900 | 1.05E-11 |
| 1200 | 9.12E-12 | 8.68E-12 | 8.67E-12 | 8.83E-12 | 6.7E-12 | 2.64E-12 | 1000 | 1.51E-11 |
| 1500 | 1.72E-11 | 1.65E-11 | 1.64E-11 | 1.66E-11 |  | 5.51E-12 | 1200 | 2.74E-11 |
| 1800 | 2.79E-11 | 2.67E-11 | 2.65E-11 | 2.67E-11 |  | 9.71E-12 | 1500 | 5.31E-11 |
| 2000 | 3.63E-11 | 3.48E-11 | 3.45E-11 | 3.47E-11 |  | 1.19E-11 | 1800 | 8.73E-11 |
| 2500 | 6.16E-11 | 5.91E-11 | 5.86E-11 | 5.89E-11 |  | 1.95E-11 | 2000 | 1.14E-10 |

*Canonical high-pressure limit rate coefficients including Eckart Tunneling used in EGME RRKM calculations.

**Values in gray are data obtained from the Arrhenius expression at lower temperatures: *k_1_(T*)=2.44×10^−11^(cm^3^/molecules) (T/298K)^0.58^×exp [(−30.26 kJ/mole/(RT)]

Table S12 Rate constants for R2: H + SiH_2_O → H_2_SiOH (in cm^3^molecule s^−1^)

| T(K) | TST | CVT | CVT/ZCT | CVT/SCT | Ref.^13,14**^ | T(K) | CVT/E* |
| --- | --- | --- | --- | --- | --- | --- | --- |
| 250 | 1.20E-16 | 1.10E-16 | 6.26E-16 | 7.60E-16 | 1.61E-15 | 200 | 5.58E-17 |
| 300 | 1.08E-15 | 9.77E-16 | 3.11E-15 | 3.53E-15 | 1.32E-14 | 300 | 1.87E-15 |
| 350 | 5.32E-15 | 4.78E-15 | 1.08E-14 | 1.19E-14 | 5.44E-14 | 400 | 1.78E-14 |
| 400 | 1.81E-14 | 1.60E-14 | 2.93E-14 | 3.14E-14 | 1.48E-13 | 500 | 8.13E-14 |
| 500 | 1.06E-13 | 9.17E-14 | 1.30E-13 | 1.36E-13 | 5.25E-13 | 600 | 2.43E-13 |
| 600 | 3.60E-13 | 3.06E-13 | 3.78E-13 | 3.89E-13 | 1.08E-12 | 700 | 5.61E-13 |
| 800 | 1.82E-12 | 2.51E-12 | 2.80E-12 | 2.85E-12 | 2.16E-12 | 800 | 1.09E-12 |
| 1000 | 5.20E-12 | 7.04E-12 | 7.53E-12 | 7.61E-12 | 2.72E-12 | 900 | 1.86E-12 |
| 1200 | 1.10E-11 | 1.47E-11 | 1.54E-11 | 1.55E-11 | 2.82E-12 | 1000 | 2.93E-12 |
| 1500 | 2.48E-11 | 3.28E-11 | 3.36E-11 | 3.38E-11 | 2.52E-12 | 1200 | 6.00E-12 |
| 1800 | 4.49E-11 | 5.87E-11 | 5.97E-11 | 5.98E-11 | 2.07E-12 | 1500 | 1.32E-11 |
| 2000 | 6.16E-11 | 8.02E-11 | 8.12E-11 | 8.14E-11 | 1.78E-12 | 1800 | 2.35E-11 |
| 2500 | 1.15E-10 | 1.48E-10 | 1.49E-10 | 1.49E-10 | 1.21E-12 | 2000 | 3.20E-11 |

*Canonical high-pressure limit rate coefficients including Eckart Tunneling used in EGME RRKM calculations.

**Values in gray are data obtained from the Arrhenius expression at lower temperatures: *k_2_(T*)=1.34×10^−8^(cm^3^/molecules) (T/298K)^-3.6^×exp [(−34.4 kJ/mole/(RT)]

Table S13 Rate constants for R3: H + SiH_2_O → H_3_SiO (in cm^3^molecule s^−1^). The rate constant values refer only to the low-level electronic structure calculation (ωB97X-D/ aug-cc-pVTZ)

| T(K) | CVT | muVT(E) | muVT(E,J) | T(K) | CVT/E* |
| --- | --- | --- | --- | --- | --- |
| 200 | 1.45E-11 | 1.33E-11 | 1.31E-11 | 200 | 1.12E-11 |
| 250 | 2.31E-11 | 2.09E-11 | 2.02E-11 | 300 | 2.72E-11 |
| 298 | 3.21E-11 | 2.89E-11 | 2.80E-11 | 400 | 4.85E-11 |
| 300 | 3.25E-11 | 2.92E-11 | 2.84E-11 | 500 | 7.40E-11 |
| 350 | 4.21E-11 | 3.82E-11 | 3.72E-11 | 600 | 1.03E-10 |
| 400 | 5.23E-11 | 4.78E-11 | 4.65E-11 | 700 | 1.36E-10 |
| 450 | 6.30E-11 | 5.75E-11 | 5.63E-11 | 800 | 1.72E-10 |
| 500 | 7.39E-11 | 6.78E-11 | 6.63E-11 | 900 | 2.10E-10 |
| 600 | 9.49E-11 | 8.95E-11 | 8.74E-11 | 1000 | 2.51E-10 |
| 700 | 1.18E-10 | 1.09E-10 | 1.09E-10 | 1200 | 3.41E-10 |
| 800 | 1.41E-10 | 1.33E-10 | 1.33E-10 | 1500 | 4.91E-10 |
| 900 | 1.64E-10 | 1.57E-10 | 1.57E-10 | 1800 | 6.59E-10 |
| 1000 | 1.88E-10 | 1.82E-10 | 1.81E-10 | 2000 | 7.80E-10 |
| 1200 | 2.09E-10 | 2.31E-10 | 2.29E-10 |  |  |
| 1300 | 2.40E-10 | 2.56E-10 | 2.53E-10 |  |  |
| 1400 | 2.69E-10 | 2.81E-10 | 2.77E-10 |  |  |
| 1500 | 2.97E-10 | 3.05E-10 | 3.01E-10 |  |  |
| 1600 | 3.24E-10 | 3.29E-10 | 3.24E-10 |  |  |
| 1800 | 3.77E-10 | 3.74E-10 | 3.71E-10 |  |  |
| 2000 | 4.28E-10 | 4.20E-10 | 4.16E-10 |  |  |
| 2400 | 5.26E-10 | 5.11E-10 | 5.00E-10 |  |  |
| 2500 | 5.50E-10 | 5.33E-10 | 5.20E-10 |  |  |

*Canonical high-pressure limit rate coefficients including Eckart Tunneling used in EGME RRKM calculations.

Table S14 Rate constants for R31: H + SiH_2_O → H_3_SiO (reactant well) → H_2_+HSiO (in cm^3^molecule s^−1^). The rate constant values refer only to the low-level electronic structure calculation (ωB97X-D/ aug-cc-pVTZ)

| T(K) | TST | CVT | CVT/ZCT | CVT/SCT | T(K) | CVT/E* |
| --- | --- | --- | --- | --- | --- | --- |
| 200 | 3.66E-12 | 3.22E-12 | 7.33E-12 | 8.70E-12 | 200 | 1.01E-21 |
| 250 | 4.93E-12 | 4.45E-12 | 7.76E-12 | 8.83E-12 | 300 | 3.06E-11 |
| 298 | 5.97E-12 | 5.49E-12 | 8.22E-12 | 9.08E-12 | 400 | 1.79E-05 |
| 300 | 6.01E-12 | 5.53E-12 | 8.24E-12 | 9.10E-12 | 500 | 6.09E-02 |
| 350 | 6.95E-12 | 6.49E-12 | 8.74E-12 | 9.45E-12 | 600 | 1.46E+01 |
| 400 | 7.80E-12 | 7.34E-12 | 9.25E-12 | 9.85E-12 | 700 | 7.50E+02 |
| 450 | 8.58E-12 | 8.13E-12 | 9.78E-12 | 1.03E-11 | 800 | 1.46E+04 |
| 500 | 9.32E-12 | 8.88E-12 | 1.03E-11 | 1.08E-11 | 900 | 1.49E+05 |
| 600 | 1.07E-11 | 1.03E-11 | 1.14E-11 | 1.18E-11 | 1000 | 9.65E+05 |
| 700 | 1.21E-11 | 1.17E-11 | 1.26E-11 | 1.29E-11 | 1200 | 1.60E+07 |
| 800 | 1.35E-11 | 1.30E-11 | 1.39E-11 | 1.41E-11 | 1500 | 2.72E+08 |
| 900 | 1.50E-11 | 1.45E-11 | 1.52E-11 | 1.54E-11 | 1800 | 1.81E+09 |
| 1000 | 1.64E-11 | 1.59E-11 | 1.65E-11 | 1.67E-11 | 2000 | 4.69E+09 |
| 1100 | 1.79E-11 | 1.74E-11 | 1.79E-11 | 1.81E-11 |  |  |
| 1200 | 1.95E-11 | 1.89E-11 | 1.94E-11 | 1.95E-11 |  |  |
| 1300 | 2.11E-11 | 2.04E-11 | 2.09E-11 | 2.10E-11 |  |  |
| 1400 | 2.28E-11 | 2.20E-11 | 2.24E-11 | 2.26E-11 |  |  |
| 1500 | 2.45E-11 | 2.36E-11 | 2.40E-11 | 2.41E-11 |  |  |
| 1600 | 2.62E-11 | 2.53E-11 | 2.57E-11 | 2.58E-11 |  |  |
| 1700 | 2.80E-11 | 2.70E-11 | 2.73E-11 | 2.74E-11 |  |  |
| 1800 | 2.98E-11 | 2.87E-11 | 2.90E-11 | 2.91E-11 |  |  |
| 1900 | 3.17E-11 | 3.05E-11 | 3.08E-11 | 3.09E-11 |  |  |
| 2000 | 3.36E-11 | 3.23E-11 | 3.26E-11 | 3.27E-11 |  |  |
| 2100 | 3.56E-11 | 3.42E-11 | 3.44E-11 | 3.45E-11 |  |  |
| 2200 | 3.76E-11 | 3.61E-11 | 3.62E-11 | 3.63E-11 |  |  |
| 2300 | 3.96E-11 | 3.80E-11 | 3.81E-11 | 3.82E-11 |  |  |
| 2400 | 4.16E-11 | 3.99E-11 | 4.00E-11 | 4.01E-11 |  |  |
| 2500 | 4.38E-11 | 4.19E-11 | 4.20E-11 | 4.20E-11 |  |  |

*Canonical high-pressure limit rate coefficients including Eckart Tunneling used in EGME RRKM calculations.

Table S15 Rate constants for R32: H_3_SiO → H_2_SiOH (in s^−1^). The rate constant values refer only to the low-level electronic structure calculation (ωB97X-D/ aug-cc-pVTZ)

| T(K) | TST | CVT | CVT/ZCT | CVT/SCT | T(K) | CVT/E* |
| --- | --- | --- | --- | --- | --- | --- |
| 200 | 9.91E-08 | 9.89E-08 | 2.57E-02 | 2.36E-01 | 200 | 3.53E-02 |
| 250 | 1.13E-03 | 1.13E-03 | 6.75E-01 | 2.52E+00 | 300 | 1.52E+01 |
| 298 | 4.77E-01 | 4.77E-01 | 1.87E+01 | 3.83E+01 | 400 | 4.14E+03 |
| 300 | 5.89E-01 | 5.88E-01 | 2.14E+01 | 4.31E+01 | 500 | 2.29E+05 |
| 350 | 5.21E+01 | 5.20E+01 | 5.11E+02 | 7.51E+02 | 600 | 3.90E+06 |
| 400 | 1.52E+03 | 1.51E+03 | 7.48E+03 | 9.50E+03 | 700 | 3.14E+07 |
| 450 | 2.10E+04 | 2.10E+04 | 6.91E+04 | 8.14E+04 | 800 | 1.55E+08 |
| 500 | 1.73E+05 | 1.73E+05 | 4.37E+05 | 4.93E+05 | 900 | 5.44E+08 |
| 600 | 4.12E+06 | 4.12E+06 | 7.62E+06 | 8.21E+06 | 1000 | 1.50E+09 |
| 700 | 4.01E+07 | 4.00E+07 | 6.21E+07 | 6.54E+07 | 1200 | 6.99E+09 |
| 800 | 2.22E+08 | 2.21E+08 | 3.08E+08 | 3.20E+08 | 1500 | 3.32E+10 |
| 900 | 8.41E+08 | 8.40E+08 | 1.09E+09 | 1.12E+09 | 1800 | 9.47E+10 |
| 1000 | 2.45E+09 | 2.45E+09 | 3.01E+09 | 3.08E+09 | 2000 | 1.61E+11 |
| 1100 | 5.89E+09 | 5.88E+09 | 6.97E+09 | 7.11E+09 |  |  |
| 1200 | 1.22E+10 | 1.22E+10 | 1.41E+10 | 1.43E+10 |  |  |
| 1300 | 2.28E+10 | 2.27E+10 | 2.57E+10 | 2.60E+10 |  |  |
| 1400 | 3.88E+10 | 3.87E+10 | 4.29E+10 | 4.34E+10 |  |  |
| 1500 | 6.16E+10 | 6.14E+10 | 6.72E+10 | 6.79E+10 |  |  |
| 1600 | 9.23E+10 | 9.21E+10 | 9.96E+10 | 1.01E+11 |  |  |
| 1700 | 1.32E+11 | 1.32E+11 | 1.41E+11 | 1.42E+11 |  |  |
| 1800 | 1.81E+11 | 1.81E+11 | 1.92E+11 | 1.94E+11 |  |  |
| 1900 | 2.41E+11 | 2.41E+11 | 2.54E+11 | 2.56E+11 |  |  |
| 2000 | 3.12E+11 | 3.11E+11 | 3.27E+11 | 3.28E+11 |  |  |
| 2100 | 3.93E+11 | 3.92E+11 | 4.10E+11 | 4.12E+11 |  |  |
| 2200 | 4.86E+11 | 4.84E+11 | 5.04E+11 | 5.07E+11 |  |  |
| 2300 | 5.89E+11 | 5.87E+11 | 6.09E+11 | 6.12E+11 |  |  |
| 2400 | 7.03E+11 | 7.01E+11 | 7.25E+11 | 7.28E+11 |  |  |
| 2500 | 8.28E+11 | 8.25E+11 | 8.51E+11 | 8.54E+11 |  |  |
|  |  |  |  |  |  |  |

*Canonical high-pressure limit rate coefficients including Eckart Tunneling used in EGME RRKM calculations.

Table S16 Fitted parameters for all the reaction paths studied using a 4-parameter Arrhenius fitting and the Dual level CVT/SCT, except for R3, which was obtained using VRC/ *E,J*- µVT and the low level surface.

The 4-parameter Arrhenius expression is given by

$$k=A\left( \frac{T}{300} \right)^{n}e^{-\frac{E\left( T+T_{0} \right)}{R\left( T^{2}+T_{0}^{2} \right)}}$$

To improve the accuracy and avoid divergence, the fitting procedure was carried out in the logarithm form:

$$lnk=\ln\left( A \right)+nln\left( \frac{T}{300} \right)-\frac{E\left( T+T_{0} \right)}{R\left( T^{2}+T_{0}^{2} \right)}$$

|  | R1 DL | R2 DL | R3LL | R31 LL | R1ap LL= (R1+R31) |
| --- | --- | --- | --- | --- | --- |
| A (cm^3^.molecule^-1^.s^-1^) | 1.20E-12 | 8.55E-13 | 7.37E-11 | 2.87E-13 | 9.33E-14 |
| n | 2.03 | 1.43763 | 0.9852 | 1.30 | 2.97 |
| E (cal.mol^-1^) | 1800.47 | 3345.30 | 497.907 | -935.42 | -1174.48 |
| T_0_ (K) | 115.75 | 142.818 | 178.648 | -48.81 | 107.89 |

Table S17 Activation energy (cal/mol) for all studied paths.

The activation energies are given by

$$E_{A}=E\frac{T^{4}+2T_{0}T^{3}-{T_{0}}^{2}T^{2}}{{(T^{2}+{T_{0}}^{2})}^{2}}+nRT$$

| T(K) | R1 DL | R2 DL | R3 LL | R31 LL | R1ap = (R1+R31) |
| --- | --- | --- | --- | --- | --- |
| 250 | 3096.43 | 4167.86 | 908.04 | 148.26 | 75.79 |
| 298 | 3410.23 | 4676.04 | 1079.08 | 195.18 | 296.20 |
| 300 | 3421.68 | 4693.39 | 1085.62 | 197.68 | 306.33 |
| 350 | 3679.95 | 5055.34 | 1236.83 | 270.41 | 575.24 |
| 400 | 3902.52 | 5318.02 | 1369.29 | 357.78 | 863.71 |
| 500 | 4299.38 | 5688.39 | 1600.26 | 558.60 | 1465.60 |
| 600 | 4673.06 | 5966.88 | 1807.72 | 779.34 | 2078.12 |
| 700 | 5042.15 | 6212.85 | 2004.75 | 1011.20 | 2691.61 |
| 800 | 5412.48 | 6448.98 | 2197.19 | 1249.86 | 3303.32 |
| 900 | 5785.77 | 6684.19 | 2387.69 | 1492.96 | 3912.62 |
| 1000 | 6162.29 | 6921.94 | 2577.52 | 1739.12 | 4519.59 |
| 1200 | 6924.04 | 7408.86 | 2957.22 | 2237.46 | 5727.57 |
| 1500 | 8083.33 | 8166.60 | 3529.20 | 2993.80 | 7528.58 |
| 1800 | 9256.08 | 8949.36 | 4104.37 | 3755.90 | 9320.96 |
| 2000 | 10042.98 | 9481.21 | 4489.27 | 4265.86 | 10512.68 |
| 2400 | 11625.00 | 10561.77 | 5261.71 | 5288.56 | 12890.97 |
| 2500 | 12021.80 | 10834.60 | 5455.26 | 5544.65 | 13484.74 |

Figure S4 Activation energy (cal/mol) for all studied paths.





Table S18. Bartis-Widom Phenomenological Rate Coefficients (in cm^3^.molecule^-1^.s^-1^).

|  |  | 500 K |  |
| --- | --- | --- | --- |
| P (atm) | H→H2SiOH | H→H3SiO | H→HSiO+H2 |
| 0.01 | 3.94E-12 | 5.24E-15 | 5.21E-12 |
| 0.1 | 1.52E-11 | 2.84E-14 | 3.88E-12 |
| 0.25 | 2.29E-11 | 4.86E-14 | 3.19E-12 |
| 0.5 | 2.97E-11 | 6.96E-14 | 2.66E-12 |
| 0.75 | 3.39E-11 | 8.43E-14 | 2.34E-12 |
| 1 | 3.69E-11 | 9.58E-14 | 2.13E-12 |
| 1.5 | 4.12E-11 | 1.13E-13 | 1.84E-12 |
| 2 | 4.42E-11 | 1.27E-13 | 1.64E-12 |
| 5 | 5.28E-11 | 1.76E-13 | 1.10E-12 |
| 10 | 5.83E-11 | 2.18E-13 | 7.86E-13 |
| 100 | 6.79E-11 | 4.12E-13 | 2.65E-13 |
|  |  | 1000 K |  |
| P (atm) | H→H2SiOH | H→H3SiO | H→HSiO+H2 |
| 0.01 | 1.97E-13 | 2.49E-15 | 1.02E-11 |
| 0.1 | 1.43E-12 | 1.22E-14 | 1.01E-11 |
| 0.25 | 2.89E-12 | 2.12E-14 | 9.96E-12 |
| 0.5 | 4.78E-12 | 3.08E-14 | 9.80E-12 |
| 0.75 | 6.33E-12 | 3.74E-14 | 9.68E-12 |
| 1 | 7.68E-12 | 4.24E-14 | 9.58E-12 |
| 1.5 | 9.99E-12 | 4.93E-14 | 9.42E-12 |
| 2 | 1.20E-11 | 5.36E-14 | 9.28E-12 |
| 5 | 2.04E-11 | 9.44E-14 | 8.74E-12 |
| 10 | 2.93E-11 | 1.37E-13 | 8.22E-12 |
| 100 | 7.37E-11 | 3.97E-13 | 5.97E-12 |
|  |  | 1500 K |  |
| P (atm) | H→H2SiOH | H→H3SiO | H→HSiO+H2 |
| 0.01 | 1.33E-14 | 6.56E-17 | 1.57E-11 |
| 0.1 | 1.20E-13 | 6.99E-16 | 1.54E-11 |
| 0.25 | 2.71E-13 | 1.85E-15 | 1.49E-11 |
| 0.5 | 4.90E-13 | 4.10E-15 | 1.42E-11 |
| 0.75 | 6.86E-13 | 6.71E-15 | 1.36E-11 |
| 1 | 8.67E-13 | 9.61E-15 | 1.32E-11 |
| 1.5 | 1.20E-12 | 1.61E-14 | 1.26E-11 |
| 2 | 1.50E-12 | 2.33E-14 | 1.22E-11 |
| 5 | 2.95E-12 | 7.65E-14 | 1.12E-11 |
| 10 | 4.71E-12 | 1.90E-13 | 1.06E-11 |
| 100 | 1.64E-11 | 2.37E-12 | 5.81E-12 |

Table S19 Kinetic isotope effect of R1/R1A (k_1_^H^/*k*_1a_^D^) and R1/R1B (k_1_^H^/k_1b_^D^) paths

|  | *k*_1_^H^/*k*_1A_^D^ | | | |  | *k*_1_^H^*/k*_1B_^D^ | | | |
| --- | --- | --- | --- | --- | --- | --- | --- | --- | --- |
| T(K) | TST | CVT | CVT/ZCT | CVT/SCT |  | TST | CVT | CVT/ZCT | CVT/SCT |
| 250 | 0.78 | 1.06 | 1.30 | 1.66 |  | 2.98 | 3.03 | 2.51 | 2.43 |
| 300 | 0.85 | 1.09 | 1.26 | 1.50 |  | 2.49 | 2.52 | 2.22 | 2.17 |
| 350 | 0.91 | 1.10 | 1.24 | 1.41 |  | 2.18 | 2.20 | 2.45 | 1.96 |
| 400 | 0.95 | 1.11 | 1.23 | 1.36 |  | 2.00 | 1.98 | 2.00 | 1.82 |
| 500 | 1.00 | 1.12 | 1.22 | 1.31 |  | 1.70 | 1.71 | 1.64 | 1.62 |
| 600 | 1.04 | 1.13 | 1.22 | 1.28 |  | 1.55 | 1.55 | 1.51 | 1.49 |
| 800 | 1.07 | 1.15 | 1.22 | 1.25 |  | 1.37 | 1.37 | 1.36 | 1.35 |
| 1000 | 1.09 | 1.15 | 1.22 | 1.23 |  | 1.29 | 1.28 | 1.27 | 1.26 |
| 1200 | 1.11 | 1.16 | 1.21 | 1.22 |  | 1.23 | 1.23 | 1.22 | 1.22 |
| 1500 | 1.12 | 1.16 | 1.21 | 1.22 |  | 1.19 | 1.18 | 1.19 | 1.19 |
| 1800 | 1.12 | 1.16 | 1.20 | 1.20 |  | 1.17 | 1.16 | 1.16 | 1.16 |
| 2000 | 1.12 | 1.17 | 1.20 | 1.20 |  | 1.16 | 1.15 | 1.16 | 1.15 |
| 2500 | 1.13 | 1.17 | 1.19 | 1.20 |  | 1.14 | 1.13 | 1.14 | 1.13 |

Cartesian coordinates (Å), electronic energies (E_e_) (a.u.), zero-point corrected energies (E_0_) (a.u.) of H, H_2_, HSiO, H_2_SiO, and the transition state (TS) computed with the M06-2X, ωB97-XD, CCSD(T) methods and with the aug-cc-pVTZ and aug-cc-pVQZ basis sets.

***M06-2X/aug-cc-pVTZ***

**H**

E_e_= −0.4982065

**H_2_**

H 0.000000 0.000000 0.369301

H 0.000000 0.000000 -0.369301

E_e_ = −1.1635613

E_0_ = −1.1533660

**HSiO**

Si 0.007198 0.000000 0.008383

O -0.000307 0.000000 1.530692

H 1.318543 0.000000 -0.751972

E_e_ = −365.2559796

E_0_ = −365.246891

**H_2_SiO**

Si 0.000000 0.000000 0.000000

O 0.000000 0.000000 1.517220

H 1.219998 0.000000 -0.828797

H -1.219565 0.000000 -0.826479

E_e_ = −365.2559796

E_0_ = −365.2468910

**TS1**

Si 0.061603 0.000000 0.057152

H -0.144014 0.000000 1.591823

H 1.502899 0.000000 -0.290416

O -1.061756 0.000000 -0.959791

H -0.219504 0.000000 2.898015

E_e_ = − 366.381905

E_0_ = − 366.364186

***ωB97X-D/aug-cc-pVTZ***

**H**

E_e_ = -0.5028031

**H_2_**

H 0.000000 0.000000 0.371513

H 0.000000 0.000000 -0.371513

E_e_ = -1.1766497

E_0_ = -1.1665480

**HSiO**

O 0.006146 0.000000 0.127423

Si 0.132387 0.000000 1.648956

H 1.528938 0.000000 2.263115

E_e_ = −365.2725152

E_0_ = −365.263877

**H_2_SiO**

O 0.000668 0.000000 1.520601

Si 0.000104 0.000000 0.001479

H 1.226256 0.000000 -0.830556

H -1.226595 0.000000 -0.829581

E_e_ = −365.9120507

E_0_ = −365.893689

**TS1**

O 1.144711 -0.302953 0.000000

Si -0.284169 0.212520 0.000000

H -0.655893 1.651971 0.000000

H -1.538198 -0.687833 0.000000

H -2.695631 -1.422733 0.000000

E_e_ = −366.4076377

E_0_ = −366.389884

**TS2**

Si -0.02510944 -0.56022192 0.00000000

O 0.20744546 1.03329820 0.00000000

H -0.13116072 -1.28749958 -1.27308022

H -0.13116072 -1.28749958 1.27308022

H -1.04553269 2.15175684 0.00000000

E_e_ = −366.405739

E_0_ = −366.386347

**H_2_SiOH**

O 0.20744546 1.03329820 0.00000000

Si -0.02510944 -0.56022192 0.00000000

H -0.13116072 -1.28749958 -1.27308022

H -0.13116072 -1.28749958 1.27308022

H -1.04553269 2.15175684 0.00000000

E_e_ = − 366.516063

E_0_ = − 366.487262

**TS3**

Si -0.353114 -0.227093 0.000000

O 1.104583 0.203812 0.000000

H -1.146357 -0.476968 -1.226568

H -1.146357 -0.476968 1.226568

H -1.602220 2.505264 0.000000

E_e_ = −366.414253

E_0_ = −366.395274

**H_3_SiO**

Si 0.019645010273 -0.034269228761 -0.046569616810

O -0.041176611512 0.073113061452 1.617702985973

H 1.411639819712 -0.014164113703 -0.555851261448

H -0.690349437018 -1.233387289008 -0.551969104200

H -0.699758781455 1.208707570020 -0.439030032068

E_e_ = −366.473061

E_0_ = −366.447326

**TS31**

Si 0.38008 0.01304 -0.19417

O -1.08553 -0.36331 0.03875

H 1.46291 -1.10242 -0.46394

H 1.14289 1.23296 0.10628

H 1.49455 -1.04289 0.56881

E_e_: -366.417416

E_0_: -366.394952

**TS32**

Si 0.49827 -0.00002 0.03601

O -1.11422 0.00002 -0.13523

H 1.26963 1.22237 -0.29581

H 1.27037 -1.22170 -0.29655

H -0.60199 -0.00058 1.17002

E_e_: -366.441712

E_0_: - 366.418353

**TS21**

Si -0.33905 -0.16764 -0.34205

O 1.23828 -0.15183 -0.15850

H -0.31978 1.38975 0.44876

H -1.18265 -0.91008 0.66150

H 0.54863 1.00784 0.40374

E_e_: -366.414183

E_0_: -366.390757

***CCSD(T)/aug-cc-pVTZ***

**H (Reac1)**

HF/ aug-cc-pVTZ E_e_ = -0.4998212

HF/ aug-cc-pV5Z E_e_ = -0.4999948

**H_2_ (Prod1)**

H 0.000000 0.000000 0.371378

H 0.000000 0.000000 -0.371378

E_e_: -1.1726356

E_0_: -1.162600

CCSD(T)/ aug-cc-pV5Z//CCSD(T)/ aug-cc-pVTZ: E_e_ = −1.1742509

T1 Diagnostic = 0.00575321

**HSiO (Prod1)**

O 0.000000 0.000000 0.000000

Si 0.000000 0.000000 1.578105

H 1.341344 0.000000 2.331950

Ee: -364.7420165

E_0_: -364.7335550

CCSD(T)/ aug-cc-pV5Z//CCSD(T)/ aug-cc-pVTZ: E_e_ = -364.7825414

T1 Diagnostic = 0.02883251

**H_2_SiO (Reac1)**

O 0.000000 0.000000 1.000000

Si 1.535441 0.000000 1.000000

H 2.360152 0.000444 -0.230518

H 2.368710 0.000055 2.225581

Ee: -365.3795302

E_0_: -365.361152

CCSD(T)/ aug-cc-pV5Z//CCSD(T)/ aug-cc-pVTZ: E_e_ = −365.4230328

T1 Diagnostic = 0.02089275

**TS1**

Si 0.000000 0.000000 0.000000

H 0.000000 0.000000 1.567693

H 1.389618 0.000000 -0.535883

O -1.262457 -0.006999 -0.873885

H 0.081443 -0.040343 2.821821

E_e_: -365.8727542

E_0_: -365.855523

CCSD(T)/ aug-cc-pV5Z//CCSD(T)/ aug-cc-pVTZ: E_e_ = −365.9160065

T1 Diagnostic = 0.02390271

**TS2**

Si -0.53329 0.00036 0.00616

O 1.00473 -0.00274 -0.18524

H -1.33124 1.23234 0.19538

H -1.33463 -1.22803 0.20955

H 2.09405 0.01257 0.99075

E_e_ = −365.871374

E_0_ = −365.851751

CCSD(T)/ aug-cc-pV5Z//CCSD(T)/ aug-cc-pVTZ: E_e_ = −365.914695

T1 Diagnostic = 0.03239066

**H_2_SiOH (Prod2)**

Si -0.57233 0.01898 -0.10575

O 1.08124 -0.11259 0.06925

H -1.10656 1.20800 0.62341

H -1.13159 -1.23701 0.45441

H 1.60092 0.66398 -0.15135

E_e_ = −365.9778036

E_0_ = −365.948939

CCSD(T)/ aug-cc-pV5Z//CCSD(T)/ aug-cc-pVTZ: E_e_ = −366.0199364

T1 Diagnostic = 0.01511290

**TS3**

No minimum – downhill from association

**H_3_SiO (Prod3)**

Si 0.000000 0.000000 0.000000

O 0.000000 0.000000 1.683311

H 1.377499 0.000000 -0.545614

H -0.758906 -1.150461 -0.545478

H -0.691442 1.280791 -0.300937

Ee: -365.9327486

E_0_: -365.906708

CCSD(T)/ aug-cc-pV5Z//CCSD(T)/ aug-cc-pVTZ: E_e_ = -365.9744913

T1 Diagnostic = 0.01873746
